# Identifying facilitators of and barriers to digital health literacy in pediatric rheumatic disease: A scoping review

**DOI:** 10.1371/journal.pdig.0001777

**Published:** 2026-10-15

**Authors:** Craig Eling, Alan M. Rosenberg, Jennifer N. Stinson, Maryam Mehtar, Jasmin Bhawra, Roona Sinha, Donna Goodridge

**Affiliations:** 1 Faculty of Nursing, University of Regina (Saskatoon Campus), Saskatoon, Canada; 2 Health Sciences Graduate Program, College of Medicine, University of Saskatchewan, Saskatoon, Canada; 3 Department of Pediatrics, College of Medicine, University of Saskatchewan, Saskatoon, Canada; 4 Research Institute, The Hospital for Sick Children, Toronto, Canada; 5 Lawrence Bloomberg Faculty of Nursing, University of Toronto, Toronto, Canada; 6 School of Occupational and Public Health, Toronto Metropolitan University, Toronto, Canada; 7 Department of Medicine, College of Medicine, University of Saskatchewan, Saskatoon, Canada; Harvard University, UNITED STATES OF AMERICA

## Abstract

Digital health (DH) technologies have the potential to benefit pediatric patients with rheumatic diseases and their caregivers. Digital health literacy (DHL) refers to the knowledge and skills necessary to use technology and the internet to manage and communicate about health. The aim of this scoping review is to identify facilitators of and barriers to DHL and map them to the Capability, Opportunity, and Motivation Model of Behavior (COM-B Model) for children and adolescents with rheumatic diseases and their primary caregivers. We searched MEDLINE, CINAHL, Embase, Web of Science, and ProQuest for English full-text primary research studies. Facilitators of and barriers to DHL identified were codified and mapped to the COM-B Model. This review adhered to the JBI scoping review framework and conformed to the Preferred Reporting Items for Systematic Reviews and Meta-Analyses (PRISMA)-Scoping Review guidelines. Of the 5789 unique records, 44 met inclusion criteria. All the included studies did not explicitly address the concept and impact of DHL. However, the studies identified promoting and detracting factors that matched the definition of DHL. Thus, these were coded as facilitators of and barriers to DHL. The COM-B Model domain of psychological capability emerged as a major determinant of DHL. The COM-B Model domain of psychological capability emerged as a prominent source of facilitators of and barriers to DHL. The design, function, language, and presentation of DH crafted to meet the developmental and educational needs of the user were essential to improving DHL. Likewise, providers fostering quality communication and answering medical and technical questions arising from DH use fostered DHL in the domain of physical opportunity. Addressing DHL requires consideration across clinical, administrative, design, and research contexts. Recognizing low DHL and enacting educational interventions are essential to empowering digital health users.

Trial Registration

Open Science Framework (https://osf.io/g4jbh/).

## Author summary

Digital health is the use of digital information and communication technologies to manage health care. The skills, know-how, and ability to get benefit from using digital health is called digital health literacy (DHL). Having lower levels of DHL means a person may not get the full benefit from digital health. We did a scoping review to identify factors that make DHL better and worse in young people with childhood rheumatic disease and their caregivers. A scoping review looks at what is already known about this in medical studies and groups the findings together into one study. Once we found the factors that make DHL better and worse, we matched them to the COM-B Model of Behavior. Using the COM-B Model helps us understand how these factors make people act. We found that how well people understand the digital health was a major reason their DHL got better or worse. We also found that people need certain things in digital health to make their DHL better. These include the app being easy to use and understand. Doctors and nurses help by telling their patients about digital health. They can also help by training their patients to use digital health and answering any questions they have.

## 1. Introduction

Digital health (DH) uses various technologies, platforms, devices, and communication methods to manage and enhance the health of individuals, groups, families, and populations [1,2]. Integrating DH into health care can provide more timely, cost-effective, and convenient healthcare services, and has the potential to address disparities and inequalities associated with sex, gender, disabilities, rural and remote isolation, and mental health challenges [1,3–5].

Pediatric rheumatic diseases (PRD) are a class of systemic inflammatory disorders that include an array of musculoskeletal and connective tissue diseases, with juvenile idiopathic arthritis (JIA) being the most common [6]. PRD can negatively impact multiple aspects of a child’s life including compromising growth and development, quality of life, social interactions, and school performance [6,7]. DH can provide reassurance and support to those with PRD and their families, improving access to rheumatology care services, and fostering self-management skills [5,8]. Furthermore, DH offers an alternative to reduce the burdens of travel for in-person health care [9].

To gain benefit from DH, users require an adequate level of digital health literacy (DHL). DHL is a set of skills and knowledge necessary to search for, access, appraise, utilize, and integrate the health information or service provided through DH [5,10–15]. While DH presents a possible solution to disparities in health care for the PRD population, it has the potential to be a disparity itself if an individual user’s DHL is not considered prior to integrating DH services into clinical practice [10,11,16–18].

As DH emerges as an integral component of modern healthcare, it is important to be able to gauge the level of DHL among patients. Understanding the factors that facilitate or hinder DHL is an essential step toward ensuring equitable and effective patient engagement with digital tools. A pilot literature search encompassing children and adolescents with a chronic disease and caregivers of pediatric patients with chronic diseases yielded no publications explicitly using the term DHL or its common synonyms (eHealth [electronic Health] literacy, mHealth [mobile Health] literacy). However, because elements of DHL can be discussed without specifically using the term DHL or its synonyms, a comprehensive scoping review (ScR) is necessary. This ScR aims to fill this gap by identifying relevant findings that traditional keyword searches may overlook [19]. A ScR is the most appropriate first step given the paucity of publications that have explicitly focused on DHL. Although our pilot search identified studies reporting the design, implementation, and appraisal of DH interventions for PRD, these studies rarely address the digital literacy required to use them. Furthermore, a ScR affords the opportunity to catalogue a broad array of published resources, regardless of study design. By identifying knowledge gaps and clarifying foundational concepts, a ScR provides a framework to guide future systematic reviews and the development of rationally conceived interventions [19].

This ScR sought to answer the two questions: 1) What are the facilitators of and barriers to DHL identified in the literature for children and adolescents with PRD and/or primary caregivers of children and adolescents with PRD? and 2) How do the facilitators of and barriers to DHL map onto the Capability, Opportunity, and Motivation Model of Behavior (COM-B Model)? Facilitators of DHL are any individual, population, or system-level factors that improve or promote an individual’s DHL. Barriers to DHL are the same but detract from an individual’s DHL. Primary caregivers is an inclusive term used to identify the person(s) who provides most of the medical care and day-to-day needs for the child and adolescent with PRD, regardless of biological relationship. Table 1 provides the population, concept, and context framework used in this study [19].

**Table 1:** Population, concept, and context framework.

Population	Context
Children (aged 0- to 9-years) and adolescents (aged 10- to 19-years) with PRD. Primary caregivers of children and adolescents with PRD [27].	Identification of factors serving as facilitators of or barriers to DHL that can affect the delivery of PRD DH-based services. This includes virtual clinic visits, digital communication, educational supports, involvement in online communities, recommendation of specific apps, and other internet-based resources. Mapping to the COM-B Model [20] identifies behaviors that promote or demote DHL.
**Concept**	
Facilitators of and barriers to DHL and mapping them to the COM-B Model [20].	

COM-B: Capability, Opportunity, and Motivation Model of Behavior; DH: digital health; DHL: digital health literacy; PRD: pediatric rheumatic disease

The COM-B Model [20] conceptualizes an individual’s behavior resulting from the interaction of specific internal and external factors [20–23]. The COM-B Model theorizes that domains of capability and opportunity influence the domain of motivation, although all three independently influence behavior [20,21,23]. Use of the COM-B Model adds a humanistic context to the findings, situating DHL as a behavioral outcome [20,22,24–26]. This approach allows for the identification of key priorities for future person-centered interventions to improving DHL.

The COM-B Model [20] assigns behaviors into the domains and subdomains of physical capability, psychological capability, reflective motivation, automatic motivation, physical opportunity, and social opportunity. Capability refers to the knowledge, skills, stamina, and ability an individual has, both physical and psychological [20,23,28]. Opportunity refers to external factors that make the behavior possible, such as the physical resources available or the influence of society, culture, and relationships (such as social supports and societal norms) [20,23,28]. Motivation refers to the internal processes that influence decisions and behavior [20,23,28]. Reflective motivation manifests in beliefs and intentions, through planning and decision making, while automatic motivation occurs more immediately in response to desires, emotions, impulses, and inhibitions [20,23,28].

## 2. Methods

The protocol associated with this ScR was written in accordance with the JBI ScR framework [19] and the Preferred Reporting Items for Systematic Reviews and Meta-Analyses (PRISMA)-ScR guidelines [29]. This project is registered with the Open Science Framework (https://osf.io/g4jbh/). The protocol has been previously described [30]. This ScR deviates from the protocol in that we did not conduct a quality appraisal of accessed literature. In accordance with JBI recommendations, the goal of a scoping review is to catalogue the available evidence without regard for quality [19].

### 2.1 Eligibility criteria

This ScR included quantitative, qualitative, and mixed methods studies from any health sciences, healthcare, or clinical setting. To be eligible, at least 50% of the study population had to be children (aged 0–9 years) and/or adolescents (aged 10–19 years) with PRD, and/or primary caregivers of children and adolescents with PRD. Diseases included under the umbrella of PRD were in accordance with the spectrum of diseases, their nomenclature, and diagnostic criteria delineated in the *Textbook of Pediatric Rheumatology* [6].

Studies published from January 1, 1974 onward were considered for inclusion, as this is the year that the term *health literacy* first appeared in the published literature [31]. Only studies available in full-text English were eligible for inclusion. Studies discussing any factors that promoted or improved DHL or detracted or delayed DHL were included. Papers were excluded if they did not present original data in full text format, such as opinion pieces, editorials, conference proceedings, grey literature, and analyses. Studies from complementary and alternative health fields were excluded. Full text articles that were in a language other than English or were not accessible digitally or paper-based were excluded.

### 2.2 Information sources

The search terms used were developed through input from experts in the field (AMR, JNS, DG, and CE), and through an independent review by a health sciences research librarian, following the Peer Review of Electronic Search Strategies (PRESS) guidelines [32]. The search terms were developed to identify: 1) studies involving participant DHL and DH use; 2) studies involving participants with rheumatic diseases or the caregivers of patients having a rheumatic disease; and 3) studies involving participants who were children, adolescents, or primary caregivers of children and adolescents. The electronic databases Ovid MEDLINE All (1946 to present), EBSCOhost CINAHL Plus with Full Text (1956 to present), Ovid Embase Classic + Embase (1947 to present), Clarivate Web of Science - Science Citation Index Expanded (SCI-EXPANDED) (1900 to present), and Clarivate ProQuest Dissertations & Theses Citation Index (1637 to present) were searched throughout January 2025, with the final search completed on January 21, 2025.

### 2.3 Search

The search strategies for all databases are available in the S1 Text in the Supporting Information.

### 2.4 Selection of sources of evidence

Identified articles were imported into Covidence (Veritas Health Innovation, Ltd., Melbourne, Australia, 2024), a web browser tool that facilitates organizing, screening, extracting data from studies, and collaboration. After duplicates were removed, three researchers (DG, RS, and CE) trialed the screening processes on 24 titles and abstracts, then met to review the selections and the inclusion and exclusion criteria to minimize selection bias [33]. Articles potentially suitable for inclusion underwent a full-text review by the same three researchers. Articles discussing factors relating to the skills and knowledge consistent with the definition of DHL were flagged for inclusion [10,11,13,16]. This included technology operation, internet navigation, and information searching skills; information evaluation, relevance, and reliability processing; privacy and security; digital communication about health; any factors found to promote or detract from an individual’s DHL; and any qualitative or quantitative evaluations of an individual’s DHL or the above-mentioned factors. Finally, a manual search of the reference lists of the included studies was conducted to identify further eligible studies. At all steps, consensus among the reviewers was established before proceeding.

Two pairs of studies were identified as using shared datasets, the first by Butler et al., 2024 [34,35] and the second Flood et al., 2024 [36] and Mulligan et al., 2022 [37]. To eliminate double counting [38], all data presented in both pairs of studies were counted once, except for data around facilitators and barriers, as these results differed between studies. Whenever possible, the source(s) of relevant data corresponding to these four studies will be noted in the findings. Thirteen studies from a group including a co-author on this paper (JNS) were included in this study [39–51]; they were scrutinized to ensure they did not contain data that would result in double counting.

## 3. Data extraction and analysis

### 3.1 Data charting process and open coding

A data extraction template was created and trialed within Covidence based upon the JBI Extraction Instrument Template [19] before two researchers (DG and CE) independently extracted the data. The template was updated in an iterative manner as data extraction progressed, and all changes were agreed upon by both researchers prior to implementing the changes. Ethnicity was categorized in accordance with Statistics Canada definitions [52], except for two studies [53,54] which used the term “non-White” without further definition.

Open coding was initiated by two researchers (DG and CE), who immersed themselves in the data to identify recurring similarities and themes within the included studies. The codes used in this study represent groupings of similar findings of facilitators or barriers that relate directly to the definition of DHL. The two researchers agreed on the codes prior to open coding. The codes were updated in an iterative manner as the data extraction progressed. After completing coding, the same two researchers mapped each code to the COM-B Model [20]. This step involved an intensive review of the data within each code, followed by categorizing codes into one of the six subdomains of the COM-B Model, in accordance with expert guidelines [20,28].

Throughout the open coding and code mapping, there were no substantive discrepancies between the researchers, and any minor variances were resolved through discussion. Demographic variables, which have been used as covariates in studies using the COM-B Model [20], were excluded from the COM-B Model mapping in this ScR, as these are mostly non-modifiable and not the target of behavioral interventions [25,55]. These included age, sex, income, education level, and living in a rural or remote location.

### 3.2 Data items

The data extraction template used in Covidence is provided in the S1 Data of the Supporting Information.

### 3.3 Synthesis of results

After completing the data extraction in Covidence, the results were exported as an Excel spreadsheet (Microsoft Corporation. Microsoft Excel. Redmond, WA: Microsoft Corporation; 2024) from which frequency counts and percentages of pertinent data were calculated.

## 4. Results

### 4.1 Selection of studies

Fig 1 depicts the decision-making process and screening results in the PRISMA-ScR [29] flow diagram. The PRISMA-ScR checklist is available in the S1 File PRISMA-ScR checklist Table of the Supporting Information.

**Fig 1:**
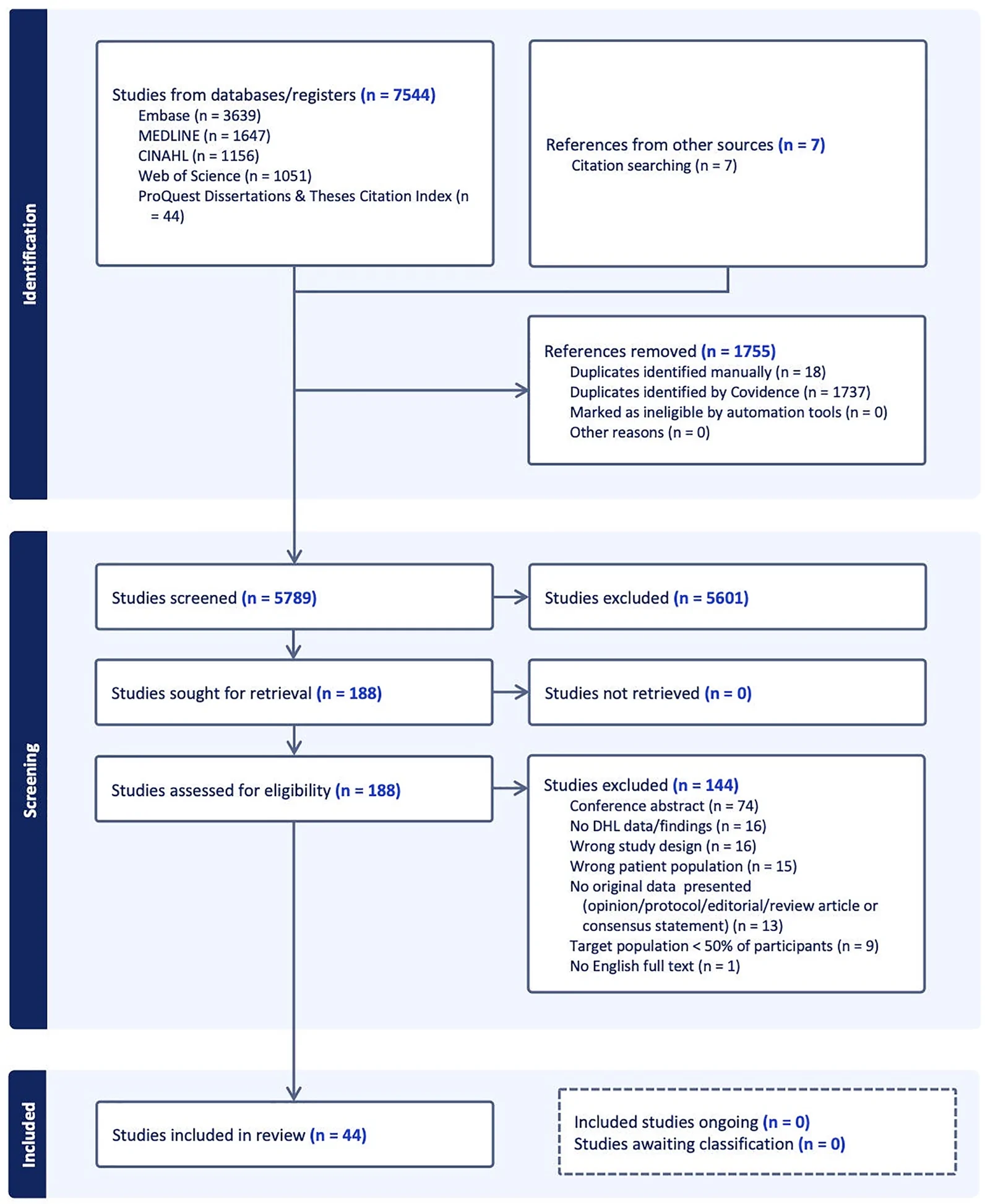
PRISMA flow diagram.

### 4.2 Characteristics of included studies

A summary of the characteristics of the studies found in this ScR are presented in Table 2. Of the 44 total studies included, 26 identified facilitators of DHL for children and adolescents, 17 identified facilitators of DHL for caregivers, 20 identified barriers to DHL for children and adolescents, and 14 identified barriers to DHL for caregivers. None of the studies used the term DHL specifically, although the reported facilitators of and barriers to utilizing DH aligned with the definition of DHL.

**Table 2:** Study characteristics.

Study characteristics	*n*= /42^*a*^(%)
**Study type**	
Data sets reported	42 (100)^*a*^
**Study done in response to the pandemic**	
Yes	4 (9.5)
**Study population**	
Children and adolescents	20 (47.6)
Children, adolescents, and caregivers	15 (35.7)
Caregivers	7 (16.7)
**Methodology used**	
Prospective longitudinal/cohort study	12 (28.6)
Qualitative research	8 (19.0)
Randomized controlled trial	6 (14.3)
Mixed methods	5 (11.9)
Cross-sectional study	5 (11.9)
Pilot study	3 (7.1)
Non-randomized experimental study	2 (4.8)
Descriptive study	1 (2.4)
**Methods of data collection**	
Questionnaires	16 (38.1)
Interviews or focus groups	13 (31.0)
Electronic messaging	3 (7.1)
Web-based education or data entry	3 (7.1)
eDiary	3 (7.1)
Appraisal of online info	1 (2.4)
Ethnographic study	1 (2.4)
Mentorship over videoconferencing	1 (2.4)
Smartphone app usage and evaluation	1 (2.4)
**Digital Data Collection Methods Described**	
Yes (fully or partially)	28 (66.7)
No	9 (21.4)
Not stated	5 (11.9)
**Multi or Single Centre Study**	
Single centre	24 (57.1)
Multicentre	18 (42.9)
**Participants with non-rheumatic diseases excluded**	
Yes	34 (81.0)
No	6 (14.3)
Not stated	2 (4.8)
**Participants with comorbidities excluded**	
No	37 (88.1)
Not stated	3 (7.1)
Yes	2 (4.8)
**Included adults with rheumatic disease**	
Yes	8 (19.0)
**Sample Size**	
< 10	3 (7.1)
11-30	13 (31.0)
31-100	11 (26.2)
101-200	9 (21.4)
201-500	4 (9.5)
501-1000	0
1001-2000	2 (4.8)

^*a*^ Both articles by Butler et al., 2024 [34,35] and both articles by Mulligan et al., 2022 [37] and Flood et al., 2024 [36] were based on the same data, thus study characteristic data was only counted once.

### 4.3 Results of individual sources of evidence

The coded facilitators of and barriers to DHL extracted from each study are shown in S1 Data in the Supporting Information.

### 4.4 Synthesis of results

For children and adolescents with PRD, 68.0% of studies identified facilitators of DHL and 50% of studies identified barriers to DHL. For caregivers of a child or adolescent with PRD, 38.6% of studies identified facilitators and 34.1% identified barriers. Double counting was not an issue for the facilitators of and barriers to DHL, as both the 2024 Butler et al. studies [34,35] and the Mulligan et al., 2022 [37] and Flood et al., 2024 [36] studies presented unique facilitators and barriers; thus, the frequencies and percentages in this section are based on all 44 studies.

Table 3 displays the facilitators of DHL mapped to the COM-B Model [20] and the demographic variables identified in the studies found in this ScR between children and adolescents and caregiver participants; Table 4 does the same, but for barriers to DHL mapped between the two populations. The percent of facilitators and barriers mapped to COM-B Model and the demographic factors is presented in Fig 2.

**Table 3:** Facilitators of DHL, by COM-B Model [20] domain and demographic variables (%).

Facilitator of DHL	Children and Adolescent participants (*n*=30/44)*^a^*	Caregiver participants (*n*=17/44)*^a^*
**Psychological capability**		
Daily social media use		5.9%
DH available in the user’s language of choice	7.7%	11.8%
DH information is written with easy-to-understand language	13.3%	11.8%
DH is appropriate to the age, developmental stage, and learning needs	43.3%	23.5%
DH platform training or technical support is available*^b,c^*	13.3%	11.8%
Familiarity with the DH technology/platform	20.0%	23.5%
Lower quality of life in children and adolescents with PRD	3.3%	
**Reflective motivation**		
Desire to learn about PRD	33.3%	5.9%
Health care provider recommended DH	6.7%	5.9%
Motivated to use DH services to manage or learn about health	13.3%	11.8%
Prefer DH services over non-DH services	10.0%	5.9%
**Automatic motivation**		
Desires social connections with others	40.0%	17.6%
DH interface is interactive, fun, and rewarding*^d^*	13.3%	
DH virtual assessment did not cause more pain or discomfort than an in-person assessment	3.3%	
**Physical opportunity**		
“Less frequent” clinic visits		5.9%
Ability to get personal health information and outputs (e.g., reminders, tracking of exercise, summaries, and reports)*^b,e^*	26.7%	5.9%
Ability to meet the time commitment of face-to-face health care services		5.9%
Ability to resolve PRD questions and concerns of that arise from using DH	3.3%	5.9%
Concerns of missing school or work for face-to-face health care visits		5.9%
Customizable DH interface and notifications*^d,e^*	16.7%	17.6%
DH data entry not burdensome	13.3%	0.0%
DH interface is user friendly*^b,c^*	40.0%	47.1%
DH platform provides data security and privacy	6.7%	
Having access to the internet and internet-capable devices*^f^*	10.0%	
History of missed face-to-face health care appointments (no-shows)		5.9%
Inability to meet the time commitment of face-to-face health care services		5.9%
Internet and technology use is convenient	13.3%	
Involved in design process of DH interface	3.3%	5.9%
Lack of access to health care and/or increased travel time/costs to health care		11.8%
**Social opportunity**		
Anonymity of the internet bypasses user inhibitions and encourages expression	3.3%	
DH platform allows for time-related flexibility to meet school or social schedule	10.0%	
Has existing social supports*^f^*	6.7%	
Patients/families have established good communication with health care providers		5.9%
Receiving peer-to-peer mentorship via DH	6.7%	
**Demographic factors**		
Age: older adolescents	10.0%	
Female PRD patients or caregivers	6.7%	5.9%
Higher education: ≥ Grade 7 for children/adolescents; ≥ post-secondary for caregivers	3.3%	5.9%
Higher income		5.9%
Living rural or remote	3.3%	5.9%

^*a*^ *Although based on the same dataset, b*oth articles by Butler et al., 2024 [34,35] and both articles by Mulligan et al., 2022 [37] and Flood et al., 2024 [36] presented different facilitators of DHL, so they were counted individually (*n* = 44). ^*b*^ Both Butler et al., 2024 [34,35] reported for children and adolescents; ^*c*^ Both Butler et al., 2024 [34,35] reported for caregivers; ^*d*^ Butler et al., 2024 *JMIR Pediatrics & Parents* [34] reported for caregivers; ^*e*^ Butler et al., 2024 *JMIR Pediatrics & Parents* [34] reported for children and adolescents; ^*f*^ Butler et al., 2024 *Healthcare* [35] reported for children and adolescents. DHL: digital health literacy; DH: digital health; PRD: pediatric rheumatic disease.

**Table 4:** Barriers to DHL, by COM-B Model [20] domain and demographic variables (%).

Barrier to DHL	Children and Adolescent participants (*n* = 21/44)^*a*^	Caregiver participants (*n* = 15/44)^*a*^
**Physical capability**		
Lower severity of PRD	4.8%	
**Psychological capability**		
Communication is more difficult or erroneous over DH	4.8%	6.7%
DH information is not written with easy-to-understand language	13.6%	6.7%
DH is appropriate to the age, developmental stage, and learning needs	28.6%	13.3%
DH platform instructions are not useful	4.8%	
Lack of familiarity with DH technology/platform^*b*^	9.5%	26.7%
Less existing PRD knowledge		6.7%
Lower confidence/effectiveness in managing child health^*c*^		6.7%
Higher quality of life in children and adolescents with PRD	4.8%	
No DH platform training or technical support is available^*d*^	4.8%	
User's DH information search strategy does not yield quality results	4.8%	
**Reflective motivation**		
Prefer non-DH services over DH services	14.3%	40.0%
Not motivated to use DH services to manage or learn about health^*d*^	4.8%	
**Automatic motivation**		
DH use is a negative reminder of PRD	14.3%	
PRD digital communication from health care providers without context/explanation causes distress^*e*^	9.5%	
Receiving or learning negative PRD information through DH is distressing	4.8%	
**Physical opportunity**		
DH interface is not user friendly^*b*^	33.3%	26.7%
DH platform does not provide data security and privacy^*d*^	14.3%	13.3%
Lack of access to health care and/or increased travel time/costs to health care		13.3%
Lack of customizable DH interface and notifications^*b,f*^	14.3%	20.0%
Lack of desirable features in DH interface	4.8%	6.7%
Limited or no access to the internet and internet-capable devices^*d*^	9.5%	20.0%
Technical issues with DH technology/platform^*d*^	14.3%	
Users find technology or notifications intrusive, annoying, or ineffective^*d*^	19.0%	
**Social opportunity**		
Anonymity of the internet increases children and adolescent dishonesty	4.8%	
DH information is not accurate or misleading	4.8%	6.7%
DH platform does not allow for time-related flexibility to meet school or social schedule^*d*^	14.3%	
Is socially disengaged/lacks social supports	19.0%	6.7%
**Demographic factors**		
Age: younger adolescents	4.8%	
Lower education: ≤ high school for caregivers		6.7%
Lower income		13.3%

^*a*^ *Although based on the same dataset, b*oth articles by Butler et al., 2024 [34,35] and both articles by Mulligan et al., 2022 [37] and Flood et al., 2024 [36] presented different barriers to DHL, so they were counted individually (*n* = 44). ^*b*^ Both Butler et al., 2024 [34,35] reported for children and adolescents; ^*c*^ Mulligan et al., 2022 [37] reported for caregivers; ^*d*^ Butler et al., 2024 *Healthcare* [35] reported for children and adolescents; ^*e*^ Butler et al., 2024 *JMIR Pediatrics & Parents* [34] reported for children and adolescents; ^*f*^ Both Butler et al., 2024 [34,35] reported for caregivers. DHL: digital health literacy; DH: digital health; PRD: pediatric rheumatic disease.

**Fig 2:**
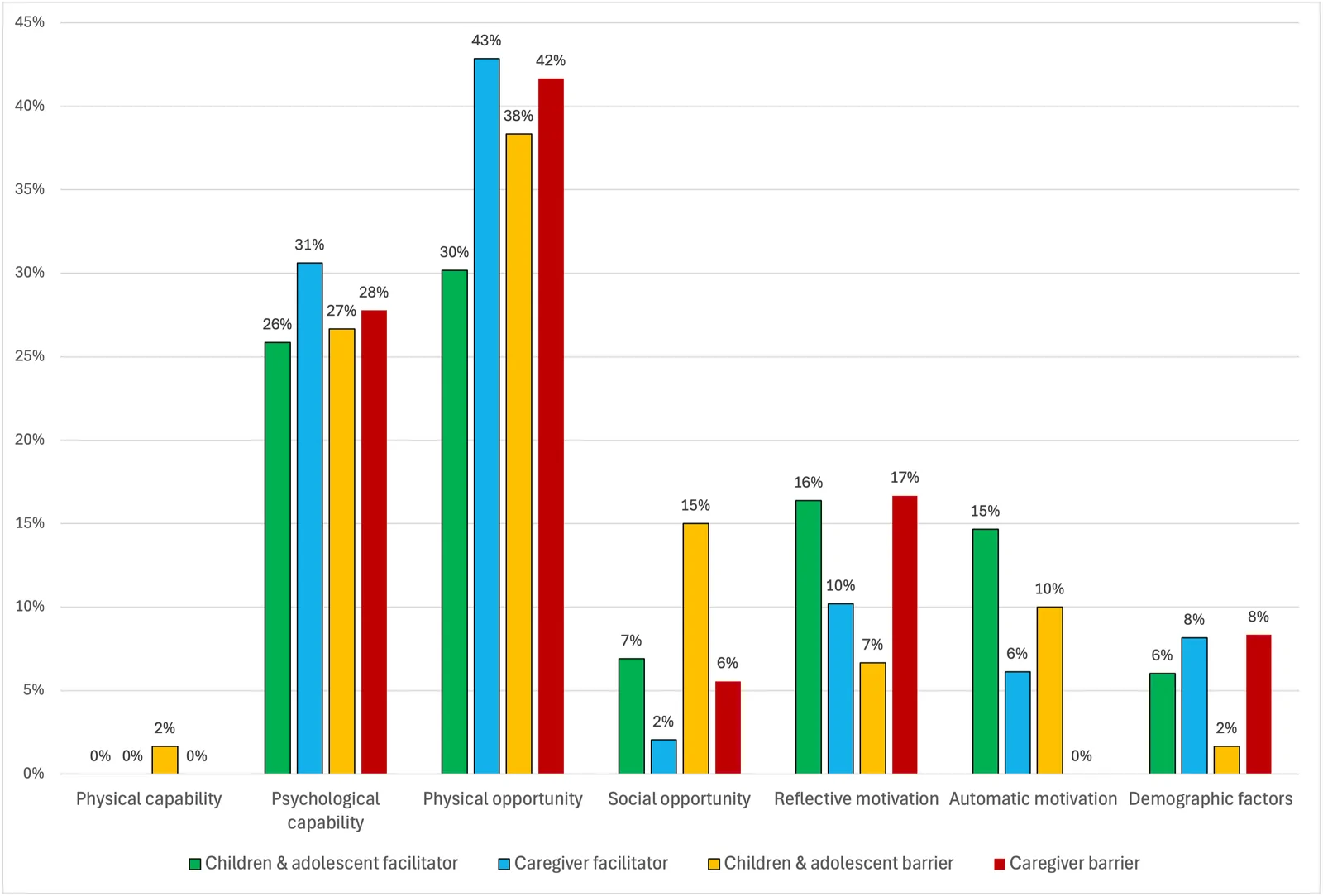
Percent of mapped to each COM-B Model [20] subdomain and the demographic factors, per facilitators and barriers per participant group.

Clinical variables, such as specific PRD diagnosis, disease course, disease duration, and duration of engagement with a pediatric rheumatology care team were extracted to gauge if a biological determinant (the diagnosis), the early (perhaps acutely stressful) or later stages of disease, and sustained engagement with health care providers over time were associated with DHL status. We found no clear indication in the studies that the disease burden (as reflected by the diagnosis), the stage of disease (early, acutely stressful phase compared to later, more chronic phases), and duration of engagement of the pediatric rheumatology care team were substantively addressed. This data from each study is shown in the S1 Data in the Supporting Information.

The sociodemographic data of sex, gender, age, ethnicity, financial data, education level, living arrangements and location, and distance/travel time to PRD clinic were extracted, when provided. Any finding of a sociodemographic factor being a facilitator of or barrier to DHL was entered as a finding in this ScR (see Tables 3 and 4). See the S1 Data in the Supporting Information for the details of data extracted from each study. The included studies did not comment on the ownership of the devices to which the children and adolescent participants had access; that is, there is no data on whether devices were shared with caregivers or if access was exclusive to the child and adolescent participants. Furthermore, in some studies the devices were loaned to the participant for the duration of the study, while other studies did not specify ownership of the device.

## 5. Discussion

As DH emerges as an integral component of modern healthcare, it is important to gauge the level of DHL among patients. Understanding the factors that facilitate or hinder DHL is an essential step toward ensuring equitable and effective patient engagement with digital tools. A surprising finding of this ScR is that of the 44 studies reporting on DH use or interventions, none explicitly addressed the influence of DHL on participant behaviors, health outcomes, or DH benefit. The lack of acknowledgement of DHL specifically in the studies included in this ScR limits the generalizability and clinical applicability of findings pertaining to DH.

Several factors might explain why DHL was not directly addressed in the literature. First, researchers may lack awareness of the concept and importance of DHL and inconsistencies in how DHL is defined [15]. Secondly, although DHL frameworks exist, such as the eHealth Literacy Framework [2] and the Lily Model [56], these are seldom applied to DHL research [15,57].

While there are definitions of DHL associated with the theoretical frameworks, the definition of DHL has evolved beyond the constraints of a single theoretical model, including the definition proposed in this study. Multiple synonyms are being used for DHL, such as *eHealth* (or *e-Health*) *literacy*, and some authors use imprecise terminology such as *digital literacy* or *health literacy* when referring to DHL [13,15]. This offers explanation to the search strategy used in this study, which utilized a robust strategy of multiple terminologies and operators to capture any studies directly or indirectly researching aspects of DHL. Finally, these three causes – the lack of DHL awareness, the absence of a conceptual consensus, and the deficiency of a standardized and cohesive definition – plausibly have a cyclical influence on each other, compounding the problem.

To address the lack of a generally accepted standardized and cohesive definition of DHL as a hypothesis generating contribution to future DHL-related research, we constructed a contemporary DHL definition derived, in part, from established conceptual frameworks [5,10–14]. Our proposed definition is: DHL is a metaliteracy comprising the ability to interface with digital platforms to find, understand, and appraise health information, to communicate about health, and to exchange information with DH services within secure, high-trust ecosystems allowing the digital fluency to resolve health concerns, advance knowledge, and sustain a healthy lifestyle. To assess the effectiveness of this definition we mapped the facilitators of and barriers to DHL to our proposed definition (see the S1 Data of the Supporting Information).

Even though we searched as far back as 1974, the year the term *health literacy* was first used [31], the first article found was published in 2006 [47]. This suggests that the use of DH in PRD, with or without clinician recommendation, is a trend only emerging over the last 20 years. However, the most recently published articles found in this study have yet to explicitly focus their research on DHL. This indicates a persisting knowledge gap where DHL exerts an unknown influence on DH use in PRD.

Most of the studies (88.1%), without double counting the two sets of studies using the same data set [34–37], collected data in North America [39–51,53,54,58–68] or Europe [36,37,69–78]. An additional two studies were based on one dataset collected in Australia [34,35]. This biases the results in favor of higher-income countries with established healthcare systems and health research funding models. Furthermore, most of the retrieved articles (71.4%) originate from countries where English is an official language. Regardless of the influence of high-income countries, the lack of direct recognition of DHL on the use of DH within the PRD population is an enduring issue.

The predominant mapping of the facilitators of and barriers to DHL to the psychological capability domain indicates that the psychological domain is critical to ensuring that DH information is presented in easy-to-understand language that meets the learning needs and is appropriate for the user’s developmental stage, a concept supported by previous research. DH information should be customized to each person’s needs, with reading level, depth of detail, and formatting adjusted to ensure it is easy to understand and use. Offering technical support and DH training would aid in improving DHL and increasing DH use.

Some themes that intersected multiple COM-B Model [20] domains emerged from the data. A key theme is that the design and functioning of DH services represent a primary determinant of DHL for children and adolescents with PRD and their caregivers [34,35,39,40,42,43,45–47,59,62,63,65,67–69,71–73,75,76,79]. Specifically, well-designed DH services function as facilitators of DHL but their absence or poor design constitute barriers supporting the need to address these factors in the early stages of DH program development.

Another theme to emerge from COM-B Model [20] mapping was communication between PRD health care providers and children and adolescents with PRD and between PRD health care providers and caregivers [34,40–42,49,54,59,61,68,70,71,73,75,77,78,80,81]. This theme reveals facilitators of and barriers to DHL relate to the quality and effectiveness of communication from PRD health care providers. Providers must establish effective communication with PRD patients and their caregivers to enhance DHL, while ineffective and erroneous communication hinders DHL.

The final theme to emerge is the opportunity for education and intervention [34,35,39–42,45,47,49,50,53,54,59,62,65,67,69–72,75,77–79,81,82] including the provision of technical and medical support and providing opportunities to educate PRD patients and their caregivers on DH services and their use, PRD in general, effective DH information search strategies, and appraisal of information.

It was surprising to find a paucity of facilitators of and barriers to DHL within the COM-B Model [20] domain of physical capability, considering that physical complaints, such as pain, musculoskeletal debility can be prominent manifestations among children with PRD [6]. This is possibly related to the exclusion criteria of the studies, as 23 studies excluded participants with physical, cognitive, co-morbid, and mental health issues that imposed limitations on the ability to complete the requirements of the study. However, the data indicates that some studies included participants with severe PRD, so it cannot be assumed that children and adolescents with severe PRD were consistently excluded. Given this, it is possible that DH is an equalizer in the domain of capability, in that DH bypasses physical ability as a factor in accessing and receiving health care. Likewise, it is possible that the domain of physical capability was not reported because an association does not exist between this domain and the facilitators of or barriers to DHL. Further research is needed to determine either of these possible relationships.

While most of our findings were mapped to the COM-B Model [20], sociodemographic and socioeconomic factors were also found to be facilitators of or barriers to DHL. Age was both a facilitator [60,66,77] and barrier [60] for children and adolescents, while higher education was a facilitator [77]. Specifically, younger age was a barrier, while older age was a facilitator. These age and education level findings were expected, as pediatric physical, cognitive, social, and communicative development would be proportional to higher levels of DHL [83]. Higher education and income levels were facilitators for caregivers [79], while lower education levels [79] and lower income [79,82] were caregiver DHL barriers. Female sex [41,65] was a facilitator for both children and adolescents [41,77] and caregivers [65], also reflecting what is generally known [84,85]. Data from the included studies’ conclusions do not allow for inferences to be made about disease characteristics, participant characteristics, child-caregiver dynamics, caregiver roles, or care settings.

A noteworthy and unexpected finding among the sociodemographic factors was that living in a rural or remote location was a facilitator for children and adolescents [69] and caregivers [81], which contrasts with the known health disparity of living in rural and remote locations [85]. Individuals in rural and remote areas may rely more heavily on DH because of geographic barriers, to access specialized care, and to reduce travel-related time and expense [86]. However, rural residency could be a barrier if access to high-speed internet technology is limited in rural/remote locations [86]. In this scoping review, only seven unique studies (16.7% without double counting [34–37] the studies using the same data set) [49,53,54,64,69,79,82] reported on rural/remote residency or travel distance to specialized clinics. Given that residency locale is underreported in the literature, the true impact of rural/remote residency on DHL is unclear and highlights the need for future research to address this knowledge gap.

## 6. Strengths and limitations

This ScR is strengthened by the comprehensive and thorough search strategy to identify relevant studies. However, this strategy can be a limitation as it interprets the study findings in a way the authors did not intend, as none of the studies directly addressed DHL. We have proposed a novel and comprehensive definition of DHL reflective of modern-day technologies and resources. The robustness of this DHL definition is supported by its effective mapping to the facilitators of and barriers to DHL we coded from the data extracted from the included studies. Our search strategy excluded grey literature, secondary articles, conference abstracts, and full text in languages other than English. Restricting the review to publications in English could potentially overlook unique cultural, economic, and epidemiological considerations relevant to DHL in a global health context and could limit the generalizability of our findings.

The rigorous approach taken, the transparent reporting of screening and data extraction, hand searching the reference sections of the included studies, use of the COM-B Model [20] to identify behavior associated with the facilitators of and barriers to DHL, and the adherence to the PRISMA-ScR guidelines [29] and JBI ScR Framework [19] are additional strengths of this ScR. The PRISMA-ScR checklist is available in the S1 PRISMA-ScR checklist Table of the Supporting Information. Additionally, the collaborative process of a multi-disciplinary team of researchers, academics, and reviewers further bolsters our results.

There was little consensus among the facilitators of and barriers to DHL found in the reviewed studies, possibly because DHL was not a prime focus in the studies’ aims and objectives. This is also likely related to the variation among the studies’ data collection dates, study designs, geography, and cultural factors. Specifically, the 44 studies collected data over a span of 19 years, from 11 different countries, with significant variation in sample size and study methodology.

Many of the studies did not report essential information, such as inclusion and exclusion criteria, recruitment methods, participant numbers, participants' sociodemographic information, PRD information, and data analysis details. Coupled with the small number of studies found (n = 44), the variation in study methodologies, sampling methods, cohort sizes, and objectives makes it difficult to compare studies.

## 7. Implications for stakeholders

While a scoping review by nature does not generate appraised evidence that can be applied to guide definitive actions, the results of our scoping review do prompt consideration of logical next actions. There is a need for researchers to develop and validate more contemporary DHL evaluation tools for children and adolescents. The utility of currently available validated DHL measurement tools is constrained, for example, by variations in pediatric developmental stages, younger child dependency on caregivers, and rapidly evolving and emerging technology [57]. In addition, these tools would be tailored to accommodate variations in age and clinical and sociodemographic contexts and account for rapidly accumulating digital information, proliferation of social media platforms, artificial intelligence, and security and privacy. These novel approaches to DHL measurement should more accurately characterize facilitators and barriers within the COM-B Model domain of opportunity [20].

Health care providers should be encouraged to incorporate consideration of DHL as part of the comprehensive evaluation of their patients and consider using validated DHL assessments tools. Furthermore, DHL should be accounted for in any study developing, implementing, or evaluating DH services for the PRD population. The knowledge gaps exposed in our scoping review should prompt researchers to undertake more thorough, prospective, longitudinal, participatory studies specifically to elucidate links between DHL levels to clinical outcomes. Our study highlights that patients and families rely on recommendations for DH resources and services. Recommending specific DH services and sources, including forums and social media accounts appropriate for different developmental stages of children, adolescents, and caregivers should be considered essential health information.

Health care administrators should acknowledge the integral and inescapable role that DH now plays in the delivery of health care and should advocate to ensure equitable access to digital resources and technologies. This includes interventions and training to improve patient and family DHL at a system-wide level. DH content managers and platform designers should be aware of the need for resources that are tailored to PRD patients and caregivers including innovations such as voice-to-text resources to accommodate patients with limited manual dexterity.

## 8. Conclusion

The findings of this ScR indicate that children and adolescents with PRD and their caregivers have specific needs, desires, and requirements around DH that are facilitators of or barriers to DHL. Without consideration for the DHL level of users, DH interventions and solutions may not provide the intended benefit and become a barrier to positive health outcomes [10,11,13,16–18]. The definition of DHL, which is mostly focused on knowledge and skills [5,10–14] aligns with the facilitators and barriers mapped to COM-B Model [20] domain of psychological capability, which encompasses skills, knowledge, and comprehension. Likewise, mapping the facilitators and barriers discovered in this ScR to the components of DHL indicates the influence of DHL on the outcomes of a study, even when not directly addressed. Therefore, even though the 44 studies did not account for participant DHL in their aims or study designs, DHL was still found to be a critical facilitator or barrier.

The results of this ScR indicate that facilitators of and barriers to DHL influenced all phases of DH program planning, design, implementation, and evaluation. Thus, the level of DHL in the intended DH user should be accounted for in the stages of program planning. This also applies to research involving DH technologies in this population, as DHL may present as a confounding variable, leading to inaccurate conclusions. The inclusion of desirable features within DH, the functioning of DH to a level appropriate to the PRD population, the provision of DH user education and exposure, and opportunity for technical and medical support are critical to facilitating DHL in children and adolescents with PRD and their caregivers. PRD care providers ought to strive to integrate effective digital communication with the PRD population as an integral component of a comprehensive care plan. Our scoping review has identified gaps in knowledge about DHL in the context of pediatric chronic diseases. Future research is required that more objectively assesses DHL participatory research involving pediatric parents and their caregivers, including task-based tools.

## Supporting information

S1 Text Search strategies and results. Contains the search strategy, terms, and results for the databases searched.
(DOCX)

S1 Data Data extracted from studies. Table 1: Contains all the data extracted from the included studies; Table 2: Data extraction template; Table 3: Coded facilitators of and barriers to DHL extracted from each study and mapped to the COM-B Model; Table 4: Country, setting, participant details, sociodemographic & economic details of included studies; Table 5: PRD diagnoses frequencies across all studies; and Table 6: Mapping the facilitators of and barriers to DHL to our novel DHL definition and the COM-B Model.
(XLSX)

S1 File PRISMA-ScR checklist. (DOCX)

## Abbreviations

DH: Digital health
PRD: Pediatric rheumatic diseases
JIA: Juvenile idiopathic arthritis
DHL: Digital health literacy
ScR: Scoping review
COM-B Model: Capability, Opportunity, and Motivation Model of Behavior
PRISMA: Preferred Reporting Items for Systematic reviews and Meta-Analyses
