# Supplementary material for: Identifying facilitators of and barriers to digital health literacy in pediatric rheumatic disease: A scoping review
Source: PLOS Digit Health. 2026 Oct 15;5(10):e0001777. doi: 10.1371/journal.pdig.0001777 (PMC13671560; doi:10.1371/journal.pdig.0001777)
Supplement: Supplementary file 1 [file pdig.0001777.s001.docx]

**Identifying Facilitators of and Barriers to Digital Health Literacy in Pediatric Rheumatic Disease: A Scoping Review**

# Craig Eling, Donna Goodridge, Jennifer N. Stinson, Maryam Mehtar, Jasmin Bhawra, Roona Sinha, Alan M. Rosenberg

# S1 File – Search Strategies

The searches utilize the controlled vocabulary thesaurus unique to each database, specifically the medical subject headings (MeSH) in MEDLINE, Emtree subject headings in Embase, subject headings in CINAHL, and Keywords Plus in Web of Sciences and ProQuest Dissertations & Theses Citation Index. Furthermore, phrase and keyword searches were also employed, using limiters and wildcard, truncation, and adjacency operators when applicable. Limiters confine the search to specific fields of the study (e.g., abstract, title, author identified keywords, synonyms, and so forth). Wildcard and truncation operators broaden a word to include alternate spellings (p?ediatric to include pediatric and paediatric) and to capture word families based upon root words (adolescen* to capture adolescent, adolescents, and adolescence).

Adjacency operators limit the search results to words or phrases to only be captured within a certain number of words to each other. For example, this is useful to limit a search using a wildcard to literac* to results within 2 words of health, thereby excluding findings of literature, literacy, and literate not associated with health. The searches strings were organized by subjects and diseases, and were combined using the Boolean operators AND, OR, and NOT.

## **Table A.** Medline search completed on January 21, 2025.

| Ovid MEDLINE(R) ALL <1946 to January 21, 2025> | | |
| --- | --- | --- |
| Search number | Query | Results from January 21, 2025 |
| 1 | exp Literacy/ or exp Health Literacy/ or exp Information Literacy/ or exp Computer Literacy/ or ((health* adj2 literac*) or (health* adj2 literate) or ((digital* or virtual*) adj3 literac*) or ((digital* or virtual*) adj3 literate)).mp. | 26,814 |
| 2 | exp telecommunication/ or exp telemedicine/ or (("eHealth*" or "e-Health*" or "emedic*" or "e-medic*" or "telehealth*" or "tele-health*" or "telemedic*" or "tele-medic*" or "mhealth*" or "m-health*" or (app? or application? or electronic or virtual* or internet or online or web or mobil* or smartphone or phone or digital* or wearable or "artificial intelligence" or robotic? or robot? or sensor* or "augmented reality" or "interactive program*")) adj3 (health* or medic*)).mp. | 324,996 |
| 3 | 1 OR 2 | 347,565 |
| 4 | exp Arthritis/ or ("juvenile idiopathic arth*" or "juvenile chronic arth*" or "juvenile rheumatoid arth*" or "p?ediatric rheum*" or "childhood arth*" or "Reiter? Syndrome" or "enthesitis related arth*" or "psoriatic arth*" or "juvenile onset still disease" or "juvenile onset still's disease" or "juvenile oligoarthritis" or "juvenile systemic arth*" or "inflammatory polyarthritis" or "rheumatic arth*" or ((polyarticular or polyarthritis) adj3 juvenile)).mp. | 320,611 |
| 5 | exp Lupus Erythematosus, Systemic/ or (lupus or neurolupus).mp. | 108,143 |
| 6 | exp vasculitis/ or (vasculitis or arteritis or endarteritis or polyarteritis or "Kawasaki* disease" or "Schoenlein-henoch* purpura" or "Henoch-schoenlein* purpura" or "Behcet* disease" or Granulomatosis or "Churg-Strauss*" or "Strauss-Churg*" or "Buerger* disease" or "Wegener* disease" or "Goodpasture* disease" or "thromboangiitis obliterans" or polyangi* or cryoglobulinemia or (ANCA adj2 vasculi*) or ((anti-neutrophil or antineutrophil) adj2 vasculi*) or (immune adj2 vasculi*)).mp. | 142,481 |
| 7 | ("ankylosing spondylitis" or "ankylosing spondylarthri*" or "rheum* spondylitis" or "bechterew* disease" or "marie-strumpell* disease" or "strumpell-marie* disease" or "spondyloarthritis ankylopoietica" or "macrophage activation syndrome" or sarcoidosis or "antiglomerular basement membrane" or "anti-glomerular basement membrane" or (enthesitis adj2 arthritis)).mp. | 59,820 |
| 8 | exp Rheumatic Diseases/ or exp Autoimmune Diseases/ or exp Rheumatic Fever/ or (rheum* or autoimmun* or auto-immun*).mp. | 899,803 |
| 9 | 4 OR 5 OR 6 OR 7 OR 8 | 1,110,057 |
| 10 | exp Child/ or exp Adolescent/ or exp Infant/ or (p?ediatric* or Infant* or child* or adolescen* or newborn* or baby or babies or teen* or juvenile or preschool* or "pre-school*" or toddler? or toddlerhood or kid or kids or youth* or boy or boys or girl or girls or "schoolchild*" or "school-child*" or "schoolage?" or "school-age?" or "school-boy?" or "schoolboy?" or "school-girl?" or "schoolgirl?" or "preteen" or "pre-teen" or teen* or "young person?" or "young people?").mp. | 5,091,434 |
| 11 | exp Family/ or exp Family Relations/ or exp Parent Child Relations/ or (parent? or carer* or caregiver or "care-giver" or father* or mother* or family or families or familial or grandparent* or "grand-parent*" or grandfather* or "grand-father*" or grandmother* or "grand-mother*" or guardian* or relativ*).mp. | 3,773,780 |
| 12 | 10 OR 11 | 7,867,712 |
| 13 | 3 AND 9 AND 12 | 1,706 |
| 14 | exp Animals/ not exp Humans/ | 5,297,693 |
| 15 | 13 NOT 14 | 1,695 |
| 16 | limit 15 to english language | 1,642 |
| 17 | limit 16 to yr="1974 -Current" | 1,640 |

## **Table B**. Embase search completed on January 21, 2025.

| Ovid Embase Classic + Embase <1947 to 2025 January 17> | | |
| --- | --- | --- |
| Search number | Query | Results from January 21, 2025 |
| 1 | exp computer literacy/ or exp health literacy/ or exp information literacy/ or exp literacy/ or exp media literacy/ or exp numeracy/ or exp telehealth/ or ((health* adj2 literac*) or (health* adj2 literate) or ((digital* or virtual*) adj3 literac*) or ((digital* or virtual*) adj3 literate)).mp. | 140,241 |
| 2 | exp telecommunication/ or exp telemedicine/ or (("eHealth*" or "e-Health*" or "emedic*" or "e-medic*" or "telehealth*" or "tele-health*" or "telemedic*" or "tele-medic*" or "mhealth*" or "m-health*" or (app? or application? or electronic or virtual* or internet or online or web or mobil* or smartphone or phone or digital* or wearable or "artificial intelligence" or robotic? or robot? or sensor* or "augmented reality" or "interactive program*")) adj3 (health* or medic*)).mp. | 426,741 |
| 3 | 1 OR 2 | 461,452 |
| 4 | exp chronic arthritis/ or exp juvenile rheumatoid arthritis/ or exp rheumatoid arthritis/ or exp arthritis/ or exp psoriatic arthritis/ or exp systemic juvenile idiopathic arthritis/ or ("juvenile idiopathic arth*" or "juvenile chronic arth*" or "juvenile rheumatoid arth*" or "p?ediatric rheum*" or "childhood arth*" or "Reiter? Syndrome" or "enthesitis related arth*" or "psoriatic arth*" or "juvenile onset still disease" or "juvenile onset still's disease" or "juvenile oligoarthritis" or "juvenile systemic arth*" or "inflammatory polyarthritis" or "rheumatic arth*" or ((polyarticular or polyarthritis) adj3 juvenile)).mp. | 675,475 |
| 5 | exp lupus erythematosus/ or (lupus or neurolupus).mp. | 192,253 |
| 6 | exp vasculitis/ or (vasculitis or arteritis or endarteritis or polyarteritis or "Kawasaki* disease" or "Schoenlein-henoch* purpura" or "Henoch-schoenlein* purpura" or "Behcet* disease" or Granulomatosis or "Churg-Strauss*" or "Strauss-Churg*" or "Buerger* disease" or "Wegener* disease" or "Goodpasture* disease" or "thromboangiitis obliterans" or polyangi* or cryoglobulinemia or (ANCA adj2 vasculi*) or ((anti-neutrophil or antineutrophil) adj2 vasculi*) or (immune adj2 vasculi*)).mp. | 247,643 |
| 7 | ("ankylosing spondylitis" or "ankylosing spondylarthri*" or "rheum* spondylitis" or "bechterew* disease" or "marie-strumpell* disease" or "strumpell-marie* disease" or "spondyloarthritis ankylopoietica" or "macrophage activation syndrome" or sarcoidosis or "antiglomerular basement membrane" or "anti-glomerular basement membrane" or (enthesitis adj2 arthritis)).mp. | 118,864 |
| 8 | exp Autoimmune Diseases/ or exp Rheumatic Fever/ or (rheum* or autoimmun* or auto-immun*).mp. | 1,204,367 |
| 9 | 4 or 5 or 6 or 7 or 8 | 1,727,601 |
| 10 | exp juvenile/ or exp pediatrics/ or (p?ediatric* or Infant* or child* or adolescen* or newborn* or baby or babies or teen* or juvenile or preschool* or "pre-school*" or toddler? or toddlerhood or kid or kids or youth* or boy or boys or girl or girls or "schoolchild*" or "school-child*" or "schoolage?" or "school-age?" or "school-boy?" or "schoolboy?" or "school-girl?" or "schoolgirl?" or "preteen" or "pre-teen" or teen* or "young person?" or "young people?").mp. | 6,052,567 |
| 11 | exp Family/ or exp Family Relations/ or exp Parent Child Relations/ or (parent? or carer* or caregiver or "care-giver" or father* or mother* or family or families or familial or grandparent* or "grand-parent*" or grandfather* or "grand-father*" or grandmother* or "grand-mother*" or guardian* or relativ*).mp. | 4,870,795 |
| 12 | 10 or 11 | 9,609,678 |
| 13 | 3 AND 9 AND 12 | 3,797 |
| 14 | exp Animals/ not exp Humans/ | 6,564,931 |
| 15 | 13 NOT 14 | 3,777 |
| 16 | exp Plants/ not exp Humans/ | 1,048,574 |
| 17 | 15 NOT 16 | 3,764 |
| 18 | limit 17 to english language | 3,716 |
| 19 | limit 18 to yr="1974 -Current" | 3,713 |

## **Table C**. CINAHL search completed on January 21, 2025.

| EBSCOhost CINAHL Plus with Full Text <1956 to 2025> | | |
| --- | --- | --- |
| Search number | Query | Results from January 21, 2025 |
| 1 | (MH "Literacy") OR (MH "Information Literacy+") OR (MH "Health Information+") OR ((health* N2 literac*) or (health* N2 literate) or ((digital* or virtual*) N2 literac*) or ((digital* or virtual*) N2 literate)) | 56,995 |
| 2 | (MH "Digital Health+") OR (MH "Telehealth+") OR (MH "Health Informatics+") OR (MH "Telecommunications+") OR (("eHealth*" or "e-Health*" or "emedic*" or "e-medic*" or "telehealth*" or "tele-health*" or "telemedic*" or "tele-medic*" or "mhealth*" or "m-health*" or (app? or application? or electronic or virtual* or internet or online or web or mobil* or smartphone or phone or digital* or wearable or "artificial intelligence" or robotic? or robot? or sensor* or "augmented reality" or "interactive program*")) N3 (health* or medic*)) | 292,173 |
| 3 | S1 OR S2 | 336,523 |
| 4 | (MH "Arthritis, Juvenile Rheumatoid") OR (MH "Arthritis, Rheumatoid+") OR (MH "Arthritis, Psoriatic") OR (MH "Reiter Disease") OR (MH "Spondylarthritis+") OR (MH "Arthritis+") OR ("juvenile idiopathic arth*" OR "juvenile chronic arth*" OR "juvenile rheumatoid arth*" OR "p#ediatric rheum*" OR pcJIA OR JIA OR JA OR JRA OR "childhood arth*" OR JCA OR "Reiter's Syndrome" OR "enthesitis related arth*" OR "psoriatic arth*" OR "juvenile onset still disease" OR "juvenile onset still's disease" OR "juvenile oligoarthritis" OR "juvenile systemic arth*" OR "inflammatory polyarthritis" OR "rheumatic arth*") OR (polyarticular OR polyarthritis N3 juvenile) | 95,933 |
| 5 | (MH "Lupus Erythematosus, Systemic+") OR (MH "Lupus Erythematosus, Cutaneous") OR (MH "Lupus Nephritis") OR ("lupus" OR "neurolupus") | 16,962 |
| 6 | (MH "Arteritis+") OR (MH "Skin Diseases, Vascular+") OR (MH "Vasculitis+") OR (MH "Vasculitis, Central Nervous System+") OR (MH "Takayasu Arteritis") OR (MH "Goodpasture's Syndrome") OR (MH "Polyarteritis Nodosa") OR (MH "Thromboangiitis Obliterans") OR (MH "Wegener's Granulomatosis") OR (MH "Macrophage Activation Syndrome") OR (MH "Behcet's Syndrome") OR (MH "Purpura, Schoenlein-Henoch") OR (MH "Churg-Strauss Syndrome") OR (vasculitis or arteritis or endarteritis or polyarteritis or "Kawasaki* disease" or "Schoenlein-henoch* purpura" or "Henoch-schoenlein* purpura" or "Behcet* disease" or Granulomatosis or "Churg-Strauss*" or "Strauss-Churg*" or "Buerger* disease" or "Wegener* disease" or "Goodpasture* disease" or "thromboangiitis obliterans" or polyangi* or cryoglobulinemia or (ANCA adj2 vasculi*) or ((anti-neutrophil or antineutrophil) N2 vasculi*) or (immune N2 vasculi*)) | 26,327 |
| 7 | (MH "Spondylarthritis+") OR (MH "Sarcoidosis") OR ("ankylosing spondylitis" or "ankylosing spondylarthri*" or "rheum* spondylitis" or "bechterew* disease" or "marie-strumpell* disease" or "strumpell-marie* disease" or "spondyloarthritis ankylopoietica" or sarcoidosis or "antiglomerular basement membrane" or "anti-glomerular basement membrane" or (enthesitis N2 arthritis)) | 16,177 |
| 8 | (MH "Rheumatic Diseases+") OR (MH "Rheumatic Fever+") OR (MH "Autoimmune Diseases+") OR (MH "Hereditary Autoinflammatory Diseases+") OR (MH "Rheumatic Heart Disease") OR (rheum* or autoimmun* or auto-immun*) | 195,842 |
| 9 | S4 OR S5 OR S6 OR S7 OR S8 | 245,587 |
| 10 | (MH "Adolescence+") OR (MH "Child+") OR (MH "Pediatrics+") OR (MH "Pediatric Care+") OR  (p#ediatric* or Infant* or child* or adolescen* or newborn* or baby or babies or teen* or juvenile or preschool* or "pre-school*" or toddler or toddlerhood or kid or youth* or boy or girl or "schoolchild*" or "school-child*" or "schoolage*" or "school-age*" or "school-boy*" or "schoolboy*" or "school-girl*" or "schoolgirl*" or "preteen" or "pre-teen" or teen* or "young person" or "young people") | 1,582,036 |
| 11 | (MH "Family+") OR (MH "Parents+") OR (MH "Family Relations+") OR (MH "Caregivers") OR  (parent or carer* or caregiver or "care-giver" or father* or mother* or family or families or familial or grandparent* or "grand-parent*" or grandfather* or "grand-father*" or grandmother* or "grand-mother*" or guardian* or relativ*) | 996,922 |
| 12 | S10 OR S11 | 2,141,342 |
| 13 | S3 AND S9 AND S12 | 1,639 |
| 14 | (MH "Animals+") NOT (MH "Human") | 92,405 |
| 15 | S13 NOT S14 | 1,638 |
| 16 | S15 AND PY 1974-2024 | 1,638 |
| 17 | S16 AND LA English | 1,584 |

## **Table D**. Web of Sciences search completed on January 21, 2025.

| Clarivate Web of Science Science Citation Index Expanded (SCI-EXPANDED) <1900 to 2025-01-19> | | |
| --- | --- | --- |
| Search number | Query | Results from January 21, 2025 |
| 1 | TS=(health* NEAR/2 literac* or health* NEAR/2 literate or digital* NEAR/3 literac* or virtual* NEAR/3 literac* or digital* NEAR/3 literate or virtual* NEAR/3 literate) | 34,986 |
| 2 | TS=(eHealth* or e-Health* or emedic* or e-medic* or telehealth* or tele-health* or telemedic* or tele-medic* or mhealth* or m-health* or telehealth* or tele-health* or telemed* or tele-med* or telecommunication* or tele-communication* or app$ NEAR/3 health* or application$ NEAR/3 health* or electronic NEAR/3 health* or virtual* NEAR/3 health* or internet NEAR/3 health* or online NEAR/3 health* or web NEAR/3 health* or mobil* NEAR/3 health* or smartphone NEAR/3 health* or phone NEAR/3 health* or digital* NEAR/3 health* or wearable NEAR/3 health* or artificial intelligence NEAR/3 health* or robotic$ NEAR/3 health* or robot$ NEAR/3 health* or sensor* NEAR/3 health* or augmented reality NEAR/3 health* or interactive program* NEAR/3 health* or app$ NEAR/3 medic* or application$ NEAR/3 medic* or electronic NEAR/3 medic* or virtual* NEAR/3 medic* or internet NEAR/3 medic* or online NEAR/3 medic* or web NEAR/3 medic* or mobil* NEAR/3 medic* or smartphone NEAR/3 medic* or phone NEAR/3 medic* or digital* NEAR/3 medic* or wearable NEAR/3 medic* or artificial intelligence NEAR/3 medic* or robotic$ NEAR/3 medic* or robot$ NEAR/3 medic* or sensor* NEAR/3 medic* or augmented reality NEAR/3 medic* or interactive program* NEAR/3 medic*) | 447,639 |
| 3 | #1 OR #2 | 477,966 |
| 4 | TS=(arthritis or juvenile idiopathic arth* or juvenile chronic arth* or juvenile rheumatoid arth* or pediatric rheum* or childhood arth* or Reiter Syndrome or Reiter’s Syndrome or enthesitis related arth* or psoriatic arth* or juvenile onset still disease or juvenile onset still's disease or juvenile oligoarthritis or juvenile systemic arth* or inflammatory polyarthritis or rheumatic arth* or polyarticular NEAR/3 juvenile or polyarthritis NEAR/3 juvenile) | 352,485 |
| 5 | TS=(lupus or neurolupus) | 143,123 |
| 6 | TS=(vasculitis or arteritis or endarteritis or polyarteritis or Kawasaki* disease or Schoenlein-henoch* purpura or Henoch-schoenlein* purpura or Behcet* or Granulomatosis or Churg-Strauss* or Strauss-Churg* or Buerger* or Wegener* or Goodpasture* or "thromboangiitis obliterans" or polyangi* or cryoglobulinemia or ANCA NEAR/2 vasculi* or anti-neutrophil NEAR/2 vasculi* or antineutrophil NEAR/2 vasculi* or immune NEAR/2 vasculi*) | 115,389 |
| 7 | TS=(ankylosing spondylitis or ankylosing spondylarthri* or rheum* spondylitis or bechterew* or marie-strumpell* or strumpell-marie* or "spondyloarthritis ankylopoietica" or "macrophage activation syndrome" or sarcoidosis or "antiglomerular basement membrane" or "anti-glomerular basement membrane" or enthesitis NEAR/2 arthritis) | 67,395 |
| 8 | TS=(rheum* or autoimmun* or auto-immun*) | 564,514 |
| 9 | #4 OR #5 OR #6 OR #7 OR #8 | 896,236 |
| 10 | TS=(p$ediatric* or Infant* or child* or adolescen* or newborn* or baby or babies or teen* or juvenile or preschool* or pre-school* or toddler or toddlerhood or kid or youth* or boy or girl or schoolchild* or school-child* or schoolage$ or school-age$ or school-boy or schoolboy or school-girl or schoolgirl or preteen or pre-teen or teen* or young person or young people) | 4,314,620 |
| 11 | TS=(parent or carer* or caregiver or care-giver or father* or mother* or family or families or familial or grandparent* or grand-parent* or grandfather* or grand-father* or grandmother* or grand-mother* or guardian* or relativ*) | 6,672,114 |
| 12 | #10 OR #11 | 9,987,585 |
| 13 | #3 AND #9 AND #12 | 1,046 |
| 14 | #13 AND PY=(1974-2024) | 1,039 |
| 15 | #14 AND LA==("ENGLISH") | 1,025 |

## **Table E**. ProQuest Dissertations & Theses Citation Index search completed on January 21, 2025.

| Clarivate ProQuest Dissertations & Theses Citation Index <1637 to 2025-01-19> | | |
| --- | --- | --- |
| Search number | Query | Results from January 21, 2025 |
| 1 | TS=(health* NEAR/2 literac* or health* NEAR/2 literate or digital* NEAR/3 literac* or virtual* NEAR/3 literac* or digital* NEAR/3 literate or virtual* NEAR/3 literate) | 4,180 |
| 2 | TS=(eHealth* or e-Health* or emedic* or e-medic* or telehealth* or tele-health* or telemedic* or tele-medic* or mhealth* or m-health* or telehealth* or tele-health* or telemed* or tele-med* or telecommunication* or tele-communication* or app$ NEAR/3 health* or application$ NEAR/3 health* or electronic NEAR/3 health* or virtual* NEAR/3 health* or internet NEAR/3 health* or online NEAR/3 health* or web NEAR/3 health* or mobil* NEAR/3 health* or smartphone NEAR/3 health* or phone NEAR/3 health* or digital* NEAR/3 health* or wearable NEAR/3 health* or artificial intelligence NEAR/3 health* or robotic$ NEAR/3 health* or robot$ NEAR/3 health* or sensor* NEAR/3 health* or augmented reality NEAR/3 health* or interactive program* NEAR/3 health* or app$ NEAR/3 medic* or application$ NEAR/3 medic* or electronic NEAR/3 medic* or virtual* NEAR/3 medic* or internet NEAR/3 medic* or online NEAR/3 medic* or web NEAR/3 medic* or mobil* NEAR/3 medic* or smartphone NEAR/3 medic* or phone NEAR/3 medic* or digital* NEAR/3 medic* or wearable NEAR/3 medic* or artificial intelligence NEAR/3 medic* or robotic$ NEAR/3 medic* or robot$ NEAR/3 medic* or sensor* NEAR/3 medic* or augmented reality NEAR/3 medic* or interactive program* NEAR/3 medic*) | 42,318 |
| 3 | 1 OR 2 | 46,096 |
| 4 | TS=(arthritis or juvenile idiopathic arth* or juvenile chronic arth* or juvenile rheumatoid arth* or pediatric rheum* or childhood arth* or Reiter Syndrome or Reiter’s Syndrome or enthesitis related arth* or psoriatic arth* or juvenile onset still disease or juvenile onset still's disease or juvenile oligoarthritis or juvenile systemic arth* or inflammatory polyarthritis or rheumatic arth* or polyarticular NEAR/3 juvenile or polyarthritis NEAR/3 juvenile) | 10,449 |
| 5 | TS=(lupus or neurolupus) | 3,132 |
| 6 | TS=(vasculitis or arteritis or endarteritis or polyarteritis or Kawasaki* disease or Schoenlein-henoch* purpura or Henoch-schoenlein* purpura or Behcet* or Granulomatosis or Churg-Strauss* or Strauss-Churg* or Buerger* or Wegener* or Goodpasture* or "thromboangiitis obliterans" or polyangi* or cryoglobulinemia or ANCA NEAR/2 vasculi* or anti-neutrophil NEAR/2 vasculi* or antineutrophil NEAR/2 vasculi* or immune NEAR/2 vasculi*) | 1,136 |
| 7 | TS=(ankylosing spondylitis or ankylosing spondylarthri* or rheum* spondylitis or bechterew* or marie-strumpell* or strumpell-marie* or "spondyloarthritis ankylopoietica" or "macrophage activation syndrome" or sarcoidosis or "antiglomerular basement membrane" or "anti-glomerular basement membrane" or enthesitis NEAR/2 arthritis) | 888 |
| 8 | TS=(rheum* or autoimmun* or auto-immun*) | 17,010 |
| 9 | #4 OR #5 OR #6 OR #7 OR #8 | 24,236 |
| 10 | TS=(p$ediatric* or Infant* or child* or adolescen* or newborn* or baby or babies or teen* or juvenile or preschool* or pre-school* or toddler or toddlerhood or kid or youth* or boy or girl or schoolchild* or school-child* or schoolage$ or school-age$ or school-boy or schoolboy or school-girl or schoolgirl or preteen or pre-teen or teen* or young person or young people) | 495,593 |
| 11 | TS=(parent or carer* or caregiver or care-giver or father* or mother* or family or families or familial or grandparent* or grand-parent* or grandfather* or grand-father* or grandmother* or grand-mother* or guardian* or relativ*) | 915,148 |
| 12 | 10 OR 11 | 1,193,944 |
| 13 | 3 AND 9 AND 12 | 75 |
| 14 | #13 AND PY=(1974-2024) | 45 |
| 15 | Refine by Language (English only) | 49 |
