# Supplementary material for: Identifying facilitators of and barriers to digital health literacy in pediatric rheumatic disease: A scoping review
Source: PLOS Digit Health. 2026 Oct 15;5(10):e0001777. doi: 10.1371/journal.pdig.0001777 (PMC13671560; doi:10.1371/journal.pdig.0001777)
Supplement: Supplementary file 3 [file pdig.0001777.s005.docx]

**PDIG-D-25-00826**

Identifying Facilitators of and Barriers to Digital Health Literacy in Pediatric Rheumatic Disease: A Scoping Review

PLOS Digital Health

**Response to editorial remarks**

Thank you very much for considering our manuscript for publication, for the thoughtful review of our work, and the opportunity to address your concerns. Our comments are provided as bullets following each editorial comment. **Please note that line and page numbers refer to the revised manuscript with track changes.**

**Journal Requirements:**

1. Please amend your detailed Financial Disclosure statement. This is published with the article. It must therefore be completed in full sentences and contain the exact wording you wish to be published.

- Lines 588-592 have been revised, and the wording matches the standard PLoS wording.

2. Please send a completed 'Competing Interests' statement, including any COIs declared by your co-authors. If you have no competing interests to declare, please state "The authors have declared that no competing interests exist". Otherwise please declare all competing interests beginning with the statement "I have read the journal's policy and the authors of this manuscript have the following competing interests:"

- Lines 593-594 have been added, and the wording matches the standard PLoS wording.

3. Please upload separate figure files in .tif or .eps format. Also, remove the figures from your manuscript file but keep the legends.

- This issue has been corrected. Figure 1 (the population, concept, and context chart) has been converted to Table 1 (lines 134-135), mainly to allow the references to automatically update.

4. We have noticed that you have uploaded Supporting Information files, but you have not included a list of legends. Please add a full list of legends for your Supporting Information files after the references list.

- Supporting Information has been updated (lines 595-603) to include the legends

5. We note that your Data Availability Statement is currently as follows: All data is available in the manuscript and supplementary appendix files.
 
 Please confirm at this time whether or not your submission contains all raw data required to replicate the results of your study. Authors must share the “minimal data set” for their submission. PLOS defines the minimal data set to consist of the data required to replicate all study findings reported in the article, as well as related metadata and methods (<https://journals.plos.org/plosone/s/data-availability#loc-minimal-data-set-definition>).

- Thank you for the opportunity to revise this. The Supporting Information has been updated. As indicated in lines 595-603, we now include:
  - **S1 File. Search strategies and results**. Contains the search strategy, terms, and results for the databases searched; “S1_file.docx”
  - **S2 File. Data extracted from studies.** Table 1: Contains all the data extracted from the included studies; Table 2: Data extraction template; Table 3: Coded facilitators of and barriers to DHL extracted from each study and mapped to the COM-B Model; Table 4: Country, setting, participant details, sociodemographic and economic details of included studies; Table 5: PRD diagnoses frequencies across all studies; and Table 6: Mapping the facilitators of and barriers to DHL to our novel DHL definition and the COM-B. “S2_file.xlsx”
  - **S3 File. PRISMA-ScR checklist.** “S3_file.docx”
  - **S4 File. PRISMA-P checklist.** “S4_file.docx””
- This is the entirety of the raw data and relevant analyses done on the data.
- References to the Supporting Information were revised throughout the manuscript.

6. Some material included in your submission may be copyrighted. According to PLOS’s copyright policy, authors who use figures or other material (e.g., graphics, clipart, maps) from another author or copyright holder must demonstrate or obtain permission to publish this material under the Creative Commons Attribution 4.0 International (CC BY 4.0) License used by PLOS journals. Please closely review the details of PLOS’s copyright requirements here: PLOS Licenses and Copyright. If you need to request permissions from a copyright holder, you may use PLOS's Copyright Content Permission form.

- Thank you for allowing us the chance to correct this. The data previously shown with a map image has been converted to a table and included in Table 4 of S2 File of the Supporting Information.

**Additional Editor Comments (if provided):**

1. Alignment with the Registered Protocol
Greater transparency is needed regarding the relationship between the published protocol and the current manuscript.
Please explicitly state whether any deviations from the registered protocol occurred. In particular, the protocol indicates that a quality appraisal tool was to be used, yet the results of any appraisal are not presented or discussed in the manuscript. If quality appraisal was conducted, summarize the findings and clarify how they informed the synthesis. If it was not conducted, please explain why and clearly note this as a deviation from the protocol.

- Thank you for this comment and for the opportunity to correct the issue. We recognize now that quality appraisal is not required for a ScR, as per JBI recommendations.
- The following was added to lines 151-153 (first paragraph of the Methods section):
  - This ScR deviates from the protocol in that we did not conduct a quality appraisal of accessed literature. In accordance with the JBI recommendations, the goal of a scoping review is to catalogue the available evidence without regard for quality [18].
- The statements of conformity to JBI ScR framework and PRISMA-ScR and the OSF registration was also moved to the top of this paragraph (deleted from lines 168-171) and the entire Methods and Data Extraction sections were moved to immediately follow the Introduction section (this is discussed further below)

Important background and rationale presented in the protocol should be incorporated into the Introduction to strengthen the justification for the scoping review. Key definitions provided in the protocol (e.g., “primary caregiver,” “facilitators,” “barriers”) should be included in the manuscript itself. As the protocol and the review function as standalone documents, these definitions should be clearly presented in the Methods section to improve transparency and reproducibility.

- Thank you for these comments and allowing us to resolve them.
- Definitions of facilitators, barriers, and primary caregivers were added to lines 119-123 of the introduction, immediately after the research questions.
  - Facilitators of and barriers to DHL are any individual, population, or system-level factors that respectively improve or detract from an individual’s DHL. Primary caregivers is an inclusive term used to identify the person(s) who provide most of the medical care and day-to-day needs for the child and adolescent with PRD, regardless of biological relationship.
- To add further clarity related to the reviewer’s concerns, the following was added to lines 175-179, under Information Sources.
  - The search terms were developed to identify: 1) studies involving participant DHL and DH use; 2) studies involving participants with rheumatic diseases or the caregivers of patients having a rheumatic disease; and 3) studies involving participants who were children, adolescents, or primary caregivers of children and adolescents.

2. Methods and Analytic Transparency
Several aspects of the Methods section require clarification and restructuring. The manuscript should follow conventional structure, with the Methods section presented immediately after the Introduction and before the Results.

- Thank you for this comment. The organization of the manuscript has been modified with sections Methods and Data Extraction and Analysis moved to after the Introduction and before the Results section. The track changes of the new location were accepted, except for the headings and subheadings, so other changes made would be clear.
- I’d like to respectfully point out that the original structure presented in the manuscript followed the Digital Health submission guidelines, which states materials and methods follows the discussion section (<https://journals.plos.org/digitalhealth/s/submission-guidelines#loc-manuscript-organization>)

Please clearly justify why a scoping review was the most appropriate methodological approach for this research question.

- Thank you for this comment. Along with the earlier comment about adding more rationale from the protocol document, we extensively revised and added lines 101-114 (paragraph preceding the research questions) to address this concern. This was added after the original two sentences of the paragraph:
  - A pilot literature search encompassing children and adolescents with a chronic disease and caregivers of pediatric patients with chronic diseases yielded no publications explicitly using the term DHL or its common synonyms (eHealth [electronic Health] literacy, mHealth [mobile Health] literacy). However, because elements of DHL can be discussed without specifically using the term DHL or its synonyms, a comprehensive scoping review (ScR) is necessary. This ScR aims to fill this gap by identifying relevant findings that traditional keyword searches may overlook [18]. A ScR is the most appropriate first step given the paucity of publications that have explicitly focused on DHL. Although our pilot search identified studies reporting the design, implementation, and appraisal of digital health interventions for PRD, these studies rarely address the digital literacy required to use them. Furthermore, a ScR affords the opportunity to catalogue a broad array of published resources, regardless of study design. By identifying knowledge gaps and clarifying foundational concepts, a ScR provides a framework to guide future systematic reviews and the development of rationally conceived interventions [18].

Provide a stronger rationale for the use of the COM-B model, including why it is suitable for synthesizing evidence related to DHL in pediatric rheumatic disease populations.

- Thank you for this recommendation.
- The paragraph introducing the COM-B (lines 125-127 and 130-132) was revised for clarity and to add the sentence (lines 129-130)
  - Use of the COM-B model adds a humanistic context to the findings, situating DHL as a behavioral outcome [19, 21, 23-25].
- The first sentence of the following paragraph (lines 136-138) was added for clarity and the second sentence revised due to the new material.

Clarify how the list of pediatric rheumatic diseases was developed, including criteria for inclusion or exclusion.

- Thank you for another relevant comment and allowing us the opportunity to remedy it. The following sentence was added to lines 158-160 (first paragraph of Eligibility Criteria):
  - Diseases included under the umbrella of PRD were in accordance with the spectrum of diseases, their nomenclature, and diagnostic criteria delineated in the *Textbook of Pediatric Rheumatology* [6].
- Lines 218-219 were deleted, as the information was redundant.

Expand the description of data extraction and synthesis procedures, particularly how facilitators and barriers were identified,…

- Thank you for the opportunity to clarify this. The following was added to lines 196-200 (first paragraph of Selection of Sources of Evidence):
  - This included technology operation, internet navigation, and information searching skills; information evaluation, relevance, and reliability processing; privacy and security; digital communication about health; any factors found to promote or detract from an individual’s DHL; and any qualitative or quantitative evaluations of an individual’s DHL or the above-mentioned factors.

…coded, and mapped to COM-B domains.

- Thank you again for the opportunity to remedy this.
- The second paragraph of the Selection of Sources of Evidence section was significantly revised. The first three sentences (lines 222-224) were removed and the following was added (lines 224-232), which includes one sentence of the original text:
  - Open coding was initiated by two researchers (DG and CE), who immersed themselves in the data to identify recurring similarities and themes within the included studies. The codes used in this study represent groupings of similar findings of facilitators or barriers that relate directly to the definition of DHL. The two researchers agreed on the codes prior to open coding. The codes were updated in an iterative manner as the data extraction progressed. After completing coding, the same two researchers mapped each code to the COM-B Model [19]. This step involved an intensive review of the data within each code, followed by categorizing codes into one of the six subdomains of the COM-B Model, in accordance with expert guidelines [19, 27].

3. Results and Interpretation
While 44 studies were included, the Results section would benefit from clearer synthesis and contextualization. Please provide a concise summary of the characteristics of included studies (e.g., countries, settings, types of digital health technologies) and of participant sociodemographic features, where available. Given the close relationship between literacy and social determinants of health, this information is important for interpreting the findings. Where possible, differentiate how facilitators and barriers vary across children, adolescents, and caregivers, and discuss whether child–caregiver dynamics influenced DHL-related factors.

**And**

Variables such as diagnosis, disease duration, and duration of rheumatology care were extracted; please briefly describe whether and how these factors shaped the findings or whether meaningful differences emerged across subgroups.

- Thank you for this thoughtful comment. There was insufficient evidence derived from this ScR to be able to generalize about these findings. Regardless, we agree this could have been addressed more appropriately. Thus, we added the following paragraph to the results section (lines 294-301):
  - Clinical variables, such as specific PRD diagnosis, disease course, disease duration, and duration of engagement with a pediatric rheumatology care team were extracted to gauge if a biological determinant (the diagnosis), the early (perhaps acutely stressful) or later stages of disease, and sustained engagement with health care providers over time were associated with DHL status. We found no clear indication in the studies that the disease burden (as reflected by the diagnosis), the stage of disease (early, acutely stressful phase compared to later, more chronic phases), and duration of engagement of the pediatric rheumatology care team were substantively addressed. See the S2 File in the Supporting Information for this data from each study.
- And the paragraph immediately after this last entry (lines 311-320):
  - The sociodemographic data of sex, gender, age, ethnicity, financial data, education level, living arrangements and location, and distance/travel time to PRD clinic were extracted, when provided. Any finding of a sociodemographic factor being a facilitator of or barrier to DHL was entered as a finding in this ScR (see tables 3 and 4). See the S2 File in the Supporting Information for the details of data extracted from each study. The included studies did not comment on the ownership of the devices to which the children and adolescent participants had access; that is, there is no data on whether devices were shared with caregivers or if access was exclusive to the child and adolescent participants. Furthermore, in some studies the devices were loaned to the participant for the duration of the study, while other studies did not specify ownership of the device.
- Discussion around these findings/lack of findings was added to lines 400-412 (last paragraph of the discussion):
  - While most of our findings were mapped to the COM-B [18], sociodemographic and socioeconomic factors were also facilitators of or barriers to DHL. Age was both a facilitator [71, 74, 75] and barrier [74] for children and adolescents, while education was a facilitator [71]. Specifically, younger age was a barrier, while older age was a facilitator. These age and education level findings were expected, as pediatric physical, cognitive, social, and communicative development would be proportional to higher levels of DHL [76]. Higher education and income levels were facilitators for caregivers [60], while lower education levels [60] and lower income [60, 73] were caregiver DHL barriers. Female sex [39, 64] was a facilitator for both children and adolescents [39, 71] and caregivers [64], also reflecting what is generally known [77, 78]. Perhaps the only surprising finding amongst the sociodemographic factors was that living in a rural or remote location was a facilitator for children and adolescents [41] and caregivers [70], which contrasts with the known health disparity of living in rural and remote locations [78]. Data from the included studies’ conclusions do not allow for inferences to be made about disease characteristics, participant characteristics, child-caregiver dynamics, caregiver roles, or care settings.

The manuscript should more clearly acknowledge that the identified facilitators and barriers are indirect and inferential, given that no study directly assessed DHL

**And**

4. Conceptual Framing and Claims About Digital Health Literacy
A major issue raised by reviewers concerns the interpretation of findings in light of the absence of direct DHL measurement.
Several statements imply conclusions about DHL itself, despite the authors’ own finding that no included study directly assessed DHL levels. These claims should be carefully revised to avoid overinterpretation.

**And**

Facilitators and barriers identified in the review should be clearly framed as hypothesized or inferred influences on DHL, rather than as demonstrated determinants.

**And**

The Discussion should more explicitly acknowledge this limitation and recalibrate claims accordingly, particularly where conclusions currently appear to reflect assumptions rather than evidence derived from measured DHL outcomes.

- Thank you for this comment; it resulted in some good discussion among the authors. We agree that the evidence gleaned from our scoping review was not sufficient to demonstrate any factor that was unequivocally a determinant of DHL. However, even though the included studies don’t use the specific term *DHL* (or synonyms) concepts and information pertinent to DHL can still be gleaned from the included studies. We believe, as originally stated, that the facilitators and barriers we identified are determinants of DHL. To add weight to this, we reinforced the definition of DHL used in our scoping review. This definition presents novel wording, although it is based upon established DHL conceptual frameworks. The definition has been added to lines 86-89 of the Introduction (lines 88-93 were removed). It reads:
  - DHL is a metaliteracy comprising the ability to interface with digital platforms to find, understand, and appraise health information, to communicate about health, and to exchange information with digital health services within secure, high-trust ecosystems allowing the digital fluency to resolve health concerns, advance knowledge, and sustain a healthy lifestyle [5, 10-14].
- This DHL definition was also added to the Discussion (lines 349-353), along with the addition of recognition of the lack of a standardized terminology and definition. Furthermore, we mapped the facilitators and barriers identified to this definition in the Supporting Information (S2 File, Table 6). This entire addition is one paragraph between lines 345-354:
  - The lack of a generally accepted standardized and cohesive definition of DHL likely further contributes to the lack of attention to DHL in the included studies. To address this, we constructed a contemporary DHL definition derived, in part, from established conceptual frameworks. Our proposed definition is: DHL is a metaliteracy comprising the ability to interface with digital platforms to find, understand, and appraise health information, to communicate about health, and to exchange information with digital health services within secure, high-trust ecosystems allowing the digital fluency to resolve health concerns, advance knowledge, and sustain a healthy lifestyle. To assess the effectiveness of our novel definition of DHL we mapped the facilitators of and barriers to DHL to our novel definition of DHL (see the S2 File of the Supporting Information).
- The novel DHL definition was discussed in the Strengths and Limitations (lines 483-486):
  - We have proposed a novel and comprehensive definition of DHL reflective of modern-day technologies and resources. The robustness of this DHL definition is supported by its effective mapping to the facilitators of and barriers to DHL we coded from the data extracted from the included studies.
- And to the conclusion (lines 552-554):
  - Likewise, mapping the facilitators and barriers discovered in this ScR to the components of DHL indicates the influence of DHL on the outcomes of a study, even if the term DHL is not explicitly used.
- We acknowledge the lack of specific use of the term DHL in lines 258-260 (first paragraph of Characteristics of Included Studies):
  - None of the studies used the term DHL specifically, although the reported facilitators of and barriers to utilizing DH aligned with the definition of DHL.
- And again in the discussion, with the addition of lines 330-334:
  - A surprising finding of this ScR is that of 44 studies reporting on DH use or interventions did not directly recognize the influence of DHL on participant behaviors, health outcomes, or DH benefit. The lack of acknowledgement of DHL specifically in the studies included in this ScR limits the generalizability and clinical applicability of findings pertaining to DH.
- With the above-mentioned additions, redundant and superseded material was removed from the discussion section: lines 334-344 and 355-358.

5. Discussion and Implications
The Discussion would benefit from deeper critical reflection and clearer translation of findings.
Please expand discussion of heterogeneity across age groups, caregiver roles, disease types, and care settings, as these factors likely shape digital health literacy needs.

- Thank you for this relevant comment and the chance to address it. This was incorporated into an addition to the discussion previously commented on regarding sociodemographic/economic and clinical factors. The addition is lines 397-411:
  - While most of our findings were mapped to the COM-B [19], sociodemographic and socioeconomic factors were also facilitators of or barriers to DHL. Age was both a facilitator [72, 75, 76] and barrier [75] for children and adolescents, while education was a facilitator [72]. Specifically, younger age was a barrier, while older age was a facilitator. These age and education level findings were expected, as pediatric physical, cognitive, social, and communicative development would be proportional to higher levels of DHL [77]. Higher education and income levels were facilitators for caregivers [61], while lower education levels [61] and lower income [61, 74] were caregiver DHL barriers. Female sex [40, 65] was a facilitator for both children and adolescents [40, 72] and caregivers [65], also reflecting what is generally known [78, 79]. Perhaps the only surprising finding amongst the sociodemographic factors was that living in a rural or remote location was a facilitator for children and adolescents [42] and caregivers [71], which contrasts with the known health disparity of living in rural and remote locations [79]. Data from the included studies’ conclusions do not allow for inferences to be made about disease characteristics, participant characteristics, child-caregiver dynamics, caregiver roles, or care settings.

Reflect more explicitly on broader structural and system-level determinants of DHL (e.g., digital access inequities, health system organization, policy context), especially given the COM-B framework.

- Thank you for this thoughtful comment. The existing literature spans diverse global regions. Therefore, generalizations from the literature about environmental and sociocultural influences on DHL is challenging. Within the COM-B framework, the domain of opportunity is specifically shaped by regional variances in digital infrastructure, health systems, and local policies. Future research into DHL should be designed to thoroughly account for regional differences in environmental, systemic, and sociocultural determinants

The restriction to English-language publications should be more clearly acknowledged as a limitation, with discussion of its implications for generalizability, particularly to low- and middle-income settings.

- We thank the reviewer for comment. We agree that excluding non-English publications is a potential limitation. We have modified the limitations section to explicitly address language restriction as a potential limitation. Line 487 was revised and we added lines 489-491:
  - Restricting the review to publications in English could potentially overlook unique cultural, economic, and epidemiological considerations relevant to DHL in a global health context and could limit the generalizability of our findings.

The implications section should be strengthened by offering more concrete guidance for clinicians, health system leaders, and digital health designers.

**AND**

Given that the absence of direct DHL measurement is a key finding, the manuscript would be substantially strengthened by proposing clear, actionable recommendations for how DHL could be measured in future research, including potential tools or developmentally appropriate assessment strategies.

- We welcome the reviewer’s suggestion to provide more specific comments about how our scoping review could be mobilized to action. We have added text to guide four audiences (care providers, health systems administrators, researchers, and digital health designers) to facilitate the translation of our findings into practical applications. We added the section Implications for Practice (lines 515-543) immediately following the Strengths and Limitations section, before the Conclusion:
  - Implications for stakeholders While a scoping review by nature does not generate appraised evidence that can be applied to guide definitive actions, the results of our scoping review do prompt consideration of logical next actions. There is a need for researchers to develop and validate more contemporary DHL evaluation tools for children and adolescents. The utility of currently available validated DHL measurement tools is constrained, for example, by variations in pediatric developmental stages, younger child dependency on caregivers, and rapidly evolving and emerging technology [80]. In addition, these tools would be tailored to accommodate variations in age and clinical and sociodemographic contexts and account for rapidly accumulating digital information, proliferation of social media platforms, artificial intelligence, and security and privacy. These novel approaches to DHL measurement should more accurately characterize facilitators and barriers within the COM-B Model domain of opportunity [19].

Health care providers should be encouraged to incorporate consideration of DHL as part of the comprehensive evaluation of their patients and consider using validated DHL assessments tools. Furthermore, DHL should be accounted for in any study developing, implementing, or evaluating DH services for the PRD population. The knowledge gaps exposed in our scoping review should prompt researchers to undertake more thorough, prospective, longitudinal, participatory studies specifically to elucidate links between DHL levels to clinical outcomes are required. Our study highlights that patients and families rely on recommendations of DH resources and services. Recommending specific DH services and sources, including forums and social media accounts appropriate different developmental stages of children, adolescents, and caregivers should be considered as essential health information.
 Health care administrators should acknowledge the integral and inescapable role that DH now plays in the delivery of health care and should advocate to ensure equitable access to digital resources and technologies. This includes interventions and training to improve patient and family DHL at a system-wide level. DH content managers and platform designers should be aware of the need for resources that are tailored to PRD patients and caregivers including innovations such as voice-to-text resources to accommodate patients with limited manual dexterity.

6. Minor and Technical Issues
Please correct the apparent error in Table 2, where a superscript “1” is present without a corresponding footnote.

- Thank you for noting this. The correction has been made.

Clarify the sentence stating that “Forty-four studies indirectly identified 260 instances of potential DHL-related facilitators or barriers,” as this phrasing is currently ambiguous.

- This sentence (lines 339-340) was removed as the discussion around the included studies failure to recognize DHL as a concept was bolstered.

Ensure consistency in terminology, spelling (e.g., British vs. American English), hyphenation, and abbreviation use throughout the manuscript.

- Thank you for this astute comment and finding these inconsistencies. They have been corrected.

Revise the abstract and conclusion to improve clarity and to ensure consistency with the study’s actual findings and limitations.

- Thank you also for this comment. Both the abstract and conclusion were updated to include all the above-mentioned changes.
- Abstract: added to the second line of the results (38-41):
  - All the included studies failed to explicitly use the term DHL. However, the studies identified factors affecting the success of DH that matched the definition of DHL. Thus, these were coded as facilitators of and barriers to DHL.
  - Added to the end of the abstract conclusion (lines 48-51): Considerations for DHL must occur in the clinical, administrative, DH design, and DH research settings. Recognition of inadequate DHL is essential and education interventions to raise the DHL of DH users is vital.
  - Other minor changes made throughout.
- As indicated above, reference to mapping the facilitators and barriers to the DHL definition was added to the conclusion (lines 552-554).
- Lines 557-559 were revised (added):
  - “… facilitators of and barriers to DHL influenced all phases of DH program planning, design, implementation, and evaluation. Thus, the level of DHL in the intended DH user should be accounted for in the…” to the first line of the second paragraph, while other content was removed.
- The last sentence was deleted, and the following was added (570-573):
  - “Our scoping review has identified gaps in knowledge about DHL in the context of pediatric chronic diseases. Future research is required to that more objectively assesses DHL participatory research involving pediatric parents and their caregivers and including task-based tools.”

**Reviewers' Comments:**

Reviewer's Responses to Questions

**Comments to the Author**

- Most of the comments have already been addressed above. Below are specific comments that were not.

The grammar and spelling were generally good. Note inconsistent hyphens - “face-to-face health care” vs. “face to face health care” “non-DH” vs. “non DH”.

**AND**

Some sentences were long and could be broken up for clarity.

- Thank you for this comment and, to the best of our knowledge, these mistakes have been corrected.

They perhaps over-use uncommon abbreviations (PRD, ScR, PCC etc.)

- Thank you for your comment. We have retained the abbreviations PRD and ScR as they occur respectively 57 and 58 times in the manuscript. However, as PCC is used only twice we have removed this abbreviation (lines 123 and 134).

It also makes this concluding statement: “The results of this ScR indicate that the intended positive outcomes of DH interventions may not be actualized if the DHL of children and adolescents with PRD and their caregiver is not accounted for at the early stages of program planning.” sound more like a pre-existing author belief or assumption than an actual finding of the scoping review data.

- Thank you for this comment. The sentence in question was deleted and replaced with (lines 557-559):
  - The results of this ScR indicate that facilitators of and barriers to DHL influenced all phases of DH program planning, design, implementation, and evaluation. Thus, the level of DHL in the intended DH user should be accounted for in the stages of program planning.

If in stating “We believe this review provides a foundational understanding for future research and informs the development of targeted interventions.” by identifying the gap in literature, this is acceptable. However, it would be more ‘useful’ to readers if these authors provided guidance on how DHL levels should be assessed in future studies.

- Thank you for this recommendation. The sentence in question was deleted and replaced with (lines 557-560):
  - Our scoping review has identified gaps in knowledge about DHL in the context of pediatric chronic diseases. Future research is required to more objectively assesses DHL participatory research involving pediatric parents and their caregivers and including task-based tools.
- Furthermore, the *Implications for stakeholders* added to the manuscript (lines 515-543) directly addresses this comment.

**Other changes**

- The ampersand removed and replaced with “and” in the author names (line 7).
- The email address for JB was removed (line 20).
- DH acronym added to lines 27, 98, in Table 4, 327.
- Lines 57-58 revised for clarity.
- Line 79 for revised for clarity.
- DHL acronym added to lines 100 and 329.
- The word “review” replaced with “ScR” in line 115.
- Terminology standardized to “JBI” throughout (line 148), including the removal of listing it within the acronyms (line 578).
- Lines 126-132 revied for clarity.
- Lines 161-163 revised for clarity.
- The author initials were revised in lines 173-174 to match authors who wish to have a middle initial in their names.
- For clarity, the number of articles screened in Covidence was revised to say “24” instead of “over 20” (line 192).
- Lines 203-206 revised for clarity.
- A sentence acknowledging the inclusion of 13 studies from one research group and that we ensured each study presented unique data added as lines 209-211.
- Line 219 revised to remove the term “race”.
- Initials removed from line 230 for clarity.
- Line 234 revised for clarity.
- For clarity the formatting of Table 1 was revised (empty rows removed, headings left justified and bolded (lines 244-245)
- The introduction and title for Figure 2 (lines 279-280 and 332-323, respectively) were revised for clarity.
- Table 3 & 4 were moved higher within the text in the manuscript
- Figure 2 was revised for clarity. The structure was changed to a vertical bar graph, which should be easier to read.
- “Digital health” changed to “DH” in line 327.
- Lines 359-360 revised for clarity.
- Line 384 revised for clarity.
- The acronym PRD was added to line 387.
- Line 384 and 395 revised for clarity.
- Line 550 revised for clarity.
- Reference numbers changed throughout the document.
