# Supplementary material for: Identifying facilitators of and barriers to digital health literacy in pediatric rheumatic disease: A scoping review
Source: PLOS Digit Health. 2026 Oct 15;5(10):e0001777. doi: 10.1371/journal.pdig.0001777 (PMC13671560; doi:10.1371/journal.pdig.0001777)
Supplement: Peer review history

# Peer review: Decision Letter 0

**DOI:** 10.1371/journal.pdig.0001777.r001

**Authors:** Mengyu Wang, Cleva Villanueva

9 Mar 2026

Response to Reviewers Revised Manuscript with Track Changes Manuscript

**Journal Requirements:**

1. Please amend your detailed Financial Disclosure statement. This is published with the article. It must therefore be completed in full sentences and contain the exact wording you wish to be published.

i. Please clarify all sources of financial support for your study. List the grants, grant numbers, and organizations that funded your study, including funding received from your institution. Please note that suppliers of material support, including research materials, should be recognized in the Acknowledgements section rather than in the Financial Disclosure.

ii. State the initials, alongside each funding source, of each author to receive each grant. For example: "This work was supported by the National Institutes of Health (####### to AM; ###### to CJ) and the National Science Foundation (###### to AM)."

iii. State what role the funders took in the study. If the funders had no role in your study, please state: “The funders had no role in study design, data collection and analysis, decision to publish, or preparation of the manuscript.”

iv. If any authors received a salary from any of your funders, please state which authors and which funders.

2. Please send a completed 'Competing Interests' statement, including any COIs declared by your co-authors. If you have no competing interests to declare, please state "The authors have declared that no competing interests exist". Otherwise please declare all competing interests beginning with the statement "I have read the journal's policy and the authors of this manuscript have the following competing interests:"

3. Please upload separate figure files in .tif or .eps format. Also, remove the figures from your manuscript file but keep the legends.

For more information about figure files please see our guidelines:

https://journals.plos.org/digitalhealth/s/figures

https://journals.plos.org/digitalhealth/s/figures#loc-file-requirements

4. We have noticed that you have uploaded Supporting Information files, but you have not included a list of legends. Please add a full list of legends for your Supporting Information files after the references list.

5. We note that your Data Availability Statement is currently as follows: All data is available in the manuscript and supplementary appendix files.

Please confirm at this time whether or not your submission contains all raw data required to replicate the results of your study. Authors must share the “minimal data set” for their submission. PLOS defines the minimal data set to consist of the data required to replicate all study findings reported in the article, as well as related metadata and methods (https://journals.plos.org/plosone/s/data-availability#loc-minimal-data-set-definition).

For example, authors should submit the following data:

- The values behind the means, standard deviations and other measures reported;

- The values used to build graphs;

- The points extracted from images for analysis.

Authors do not need to submit their entire data set if only a portion of the data was used in the reported study.

If your submission does not contain these data, please either upload them as Supporting Information files or deposit them to a stable, public repository and provide us with the relevant URLs, DOIs, or accession numbers. For a list of recommended repositories, please see https://journals.plos.org/plosone/s/recommended-repositories.

If there are ethical or legal restrictions on sharing a de-identified data set, please explain them in detail (e.g., data contain potentially sensitive information, data are owned by a third-party organization, etc.) and who has imposed them (e.g., an ethics committee). Please also provide contact information for a data access committee, ethics committee, or other institutional body to which data requests may be sent. If data are owned by a third party, please indicate how others may request data access.

6. Some material included in your submission may be copyrighted. According to PLOS’s copyright policy, authors who use figures or other material (e.g., graphics, clipart, maps) from another author or copyright holder must demonstrate or obtain permission to publish this material under the Creative Commons Attribution 4.0 International (CC BY 4.0) License used by PLOS journals. Please closely review the details of PLOS’s copyright requirements here: PLOS Licenses and Copyright. If you need to request permissions from a copyright holder, you may use PLOS's Copyright Content Permission form.

Please respond directly to this email or email the journal office and provide any known details concerning your material's license terms and permissions required for reuse, even if you have not yet obtained copyright permissions or are unsure of your material's copyright compatibility.

Potential Copyright Issues:

a. Figure A in S4 Appendix: please (a) provide a direct link to the base layer of the map (i.e., the country or region border shape) and ensure this is also included in the figure legend; and (b) provide a link to the terms of use / license information for the base layer image or shapefile. We cannot publish proprietary or copyrighted maps (e.g. Google Maps, Mapquest) and the terms of use for your map base layer must be compatible with our CC-BY 4.0 license.

Note: if you created the map in a software program like R or ArcGIS, please locate and indicate the source of the basemap shapefile onto which data has been plotted.

If your map was obtained from a copyrighted source please amend the figure so that the base map used is from an openly available source. Alternatively, please provide explicit written permission from the copyright holder granting you the right to publish the material under our CC-BY 4.0 license.

Please note that the following CC BY licenses are compatible with PLOS license: CC BY 4.0, CC BY 2.0 and CC BY 3.0, meanwhile such licenses as CC BY-ND 3.0 and others are not compatible due to additional restrictions.

If you are unsure whether you can use a map or not, please do reach out and we will be able to help you. The following websites are good examples of where you can source open access or public domain maps:

* U.S. Geological Survey (USGS) - All maps are in the public domain. (http://www.usgs.gov)

* PlaniGlobe - All maps are published under a Creative Commons license so please cite “PlaniGlobe, http://www.planiglobe.com, CC BY 2.0” in the image credit after the caption. (http://www.planiglobe.com/?lang=enl)

* Natural Earth - All maps are public domain. (http://www.naturalearthdata.com/about/terms-of-use/)

**Additional Editor Comments (if provided):**

However, after careful consideration of the reviewers’ comments, substantial revisions are required before the manuscript can be considered for publication. While the topic is relevant and the methodological approach is generally sound, several issues related to alignment with the protocol, conceptual clarity, methodological transparency, interpretation of findings, and strength of conclusions need to be addressed. Collectively, these concerns warrant a Major Revision.

Key points requiring attention are summarized below.

1. Alignment with the Registered Protocol

Greater transparency is needed regarding the relationship between the published protocol and the current manuscript.

• Please explicitly state whether any deviations from the registered protocol occurred. In particular, the protocol indicates that a quality appraisal tool was to be used, yet the results of any appraisal are not presented or discussed in the manuscript. If quality appraisal was conducted, summarize the findings and clarify how they informed the synthesis. If it was not conducted, please explain why and clearly note this as a deviation from the protocol.

• Important background and rationale presented in the protocol should be incorporated into the Introduction to strengthen the justification for the scoping review.

• Key definitions provided in the protocol (e.g., “primary caregiver,” “facilitators,” “barriers”) should be included in the manuscript itself. As the protocol and the review function as standalone documents, these definitions should be clearly presented in the Methods section to improve transparency and reproducibility.

2. Methods and Analytic Transparency

Several aspects of the Methods section require clarification and restructuring.

• The manuscript should follow conventional structure, with the Methods section presented immediately after the Introduction and before the Results.

• Please clearly justify why a scoping review was the most appropriate methodological approach for this research question.

• Provide a stronger rationale for the use of the COM-B model, including why it is suitable for synthesizing evidence related to DHL in pediatric rheumatic disease populations.

• Clarify how the list of pediatric rheumatic diseases was developed, including criteria for inclusion or exclusion.

• Expand the description of data extraction and synthesis procedures, particularly how facilitators and barriers were identified, coded, and mapped to COM-B domains.

3. Results and Interpretation

While 44 studies were included, the Results section would benefit from clearer synthesis and contextualization.

• Please provide a concise summary of the characteristics of included studies (e.g., countries, settings, types of digital health technologies) and of participant sociodemographic features, where available. Given the close relationship between literacy and social determinants of health, this information is important for interpreting the findings.

• Where possible, differentiate how facilitators and barriers vary across children, adolescents, and caregivers, and discuss whether child–caregiver dynamics influenced DHL-related factors.

• Variables such as diagnosis, disease duration, and duration of rheumatology care were extracted; please briefly describe whether and how these factors shaped the findings or whether meaningful differences emerged across subgroups.

• The manuscript should more clearly acknowledge that the identified facilitators and barriers are indirect and inferential, given that no study directly assessed DHL.

4. Conceptual Framing and Claims About Digital Health Literacy

A major issue raised by reviewers concerns the interpretation of findings in light of the absence of direct DHL measurement.

• Several statements imply conclusions about DHL itself, despite the authors’ own finding that no included study directly assessed DHL levels. These claims should be carefully revised to avoid overinterpretation.

• Facilitators and barriers identified in the review should be clearly framed as hypothesized or inferred influences on DHL, rather than as demonstrated determinants.

• The Discussion should more explicitly acknowledge this limitation and recalibrate claims accordingly, particularly where conclusions currently appear to reflect assumptions rather than evidence derived from measured DHL outcomes.

5. Discussion and Implications

The Discussion would benefit from deeper critical reflection and clearer translation of findings.

• Please expand discussion of heterogeneity across age groups, caregiver roles, disease types, and care settings, as these factors likely shape digital health literacy needs.

• Reflect more explicitly on broader structural and system-level determinants of DHL (e.g., digital access inequities, health system organization, policy context), especially given the COM-B framework.

• The restriction to English-language publications should be more clearly acknowledged as a limitation, with discussion of its implications for generalizability, particularly to low- and middle-income settings.

• The implications section should be strengthened by offering more concrete guidance for clinicians, health system leaders, and digital health designers.

• Given that the absence of direct DHL measurement is a key finding, the manuscript would be substantially strengthened by proposing clear, actionable recommendations for how DHL could be measured in future research, including potential tools or developmentally appropriate assessment strategies.

6. Minor and Technical Issues

• Please correct the apparent error in Table 2, where a superscript “1” is present without a corresponding footnote.

• Clarify the sentence stating that “Forty-four studies indirectly identified 260 instances of potential DHL-related facilitators or barriers,” as this phrasing is currently ambiguous.

• Ensure consistency in terminology, spelling (e.g., British vs. American English), hyphenation, and abbreviation use throughout the manuscript.

• Revise the abstract and conclusion to improve clarity and to ensure consistency with the study’s actual findings and limitations.

**Reviewers' Comments:**

**Comments to the Author**

1. Does this manuscript meet PLOS Digital Health’s publication criteria?

Reviewer #1: Yes

Reviewer #2: Partly

**********

2. Has the statistical analysis been performed appropriately and rigorously?

Reviewer #1: Yes

Reviewer #2: N/A

**********

3. Have the authors made all data underlying the findings in their manuscript fully available (please refer to the Data Availability Statement at the start of the manuscript PDF file)?

The PLOS Data policy

Reviewer #1: Yes

Reviewer #2: Yes

**********

4. Is the manuscript presented in an intelligible fashion and written in standard English?

Reviewer #1: Yes

Reviewer #2: Yes

**********

Reviewer #1: This scoping review examines an important and under-explored topic related to the facilitators and barriers of digital health literacy among children and adolescence with rheumatic diseases, and their caregivers. The review is strengthened by being a registered project and having a published protocol. The findings provide a valuable mapping of digital literacy needs across children, adolescents, and caregivers. However, several areas would benefit from clarification and refinement to improve methodological rigor, conceptual clarity, and interpretability.

1. Alignment with the registered project and available protocol

- The scoping review would benefit from a clearer link to the registered project and available protocol, including explicitly stating whether any deviations from the protocol occurred. For example, the protocol indicates that a quality appraisal tool was used, but the results of the appraisal are not presented or discussed in the manuscript. If quality appraisal was conducted, please summarize the findings. If not, clarify why and note any deviations.

- The protocol provides important rationale for why this scoping review is needed, but much of this context is missing from the Introduction. Consider incorporating this rationale in manuscript to strengthen the justification for the scoping review.

- Several key definitions provided in the protocol (such as “primary caregiver,” “facilitators,” and “barriers”) are not included in the manuscript. Because both the protocol and the scoping review should function as standalone documents, these definitions should also be clearly presented in the Methods section of the scoping review. Including them will improve transparency and help readers fully understand the eligibility criteria and how studies were classified.

2. Methods and analytic transparency

- Consider restructuring the manuscript so that the Methods section follows the Introduction, as is standard for peer-reviewed papers. Presenting the Results first raises several methodological questions that would be resolved by reading the Methods earlier.

To strengthen rigor and reproducibility, please provide a clearer explanation of the methodological approach, including:

- Briefly explaining why a scoping review was the most appropriate review type for this research question.

- Describing the appropriateness of using the COM-B model for this specific population, and how it guided your synthesis.

- Clarifying how the list of pediatric rheumatic diseases was developed. What criteria or decisions guided which diseases were included or excluded?

3. Results

- Although 44 studies met inclusion criteria, there is no clear summary of the sociodemographic characteristics of the populations represented. Given the strong relationship between literacy and social determinants of health, it would be helpful to know whether certain groups (e.g., rural populations, marginalized communities, low-income families) were over- or under-represented. The Results section would benefit from a concise summary of the characteristics of the included studies (e.g., country of origin, setting, or type of digital health technology), rather than relying primarily on listing the tables.

-This section would also be strengthened by clearly differentiating how digital health literacy needs vary between children, adolescents, and caregivers, and by noting whether the child-caregiver relationship is a factor that impacts the facilitators of or barriers to digital health literacy, if this information was available from the included studies.

- Because the data extraction template indicates that variables such as diagnosis, disease duration, and rheumatic care duration were captured, it would be helpful to briefly describe how these factors shaped the findings and whether any meaningful differences emerged across pediatric rheumatic disease subgroups.

4. Strengthen the discussion

- The Discussion should more explicitly address heterogeneity across patient age groups, caregiver roles, disease types, and care settings, as these factors likely influence digital health literacy needs. Given that literacy demands may also differ by the type of digital health technology used, this is an important dimension to reflect on.

- Since the synthesis is guided by the COM-B framework, it would be helpful to further elaborate on the broader structural or health system-level determinants of digital health literacy, such as inequities in digital access, health service organization, or policy contexts.

- The search appears limited to English full-text primary research. Given that digital health literacy is strongly shaped by global health systems and inequities, restricting to English-language publications may bias the findings toward higher-income settings. This should be more explicitly acknowledged as a limitation, and its implications for generalizability should be discussed.

5. Implications for policy and practice

- The manuscript makes several valuable points, and the Discussion could be further strengthened by providing a more robust discussion on the implications of this scoping review. In particular, translating the findings for clinicians, health system leaders, and digital health designers (e.g., targeted digital education supports or caregiver engagement strategies) would enhance the applied value of this work.

6. Minor and technical comments

- There appears to be an error in Table 2, where a superscript “1” is present but no footnote is provided.

- The sentence in the Discussion stating that “Forty-four studies indirectly identified 260 instances of potential DHL-related facilitators or barriers” is somewhat unclear. It may be helpful to further clarify this sentence.

Reviewer #2: The authors conducted a scoping review of 44/5789 screened studies to identify factors that facilitate or hinder digital health literacy among children and adolescents with pediatric rheumatic diseases and their caregivers. They found no studies directly measuring digital health literacy, but many reported relevant influences—most mapping to psychological capability and physical opportunity within the COM‑B behavioral framework. Overall, the review concludes that clear digital health design, appropriate supports, and effective provider communication are essential to enabling users to benefit from digital health tools. They identify “No study directly addressed DHL” which is in itself an important finding and call to action. Many of the findings otherwise seem high level and obvious. I appreciated the connection of facilitators and barriers to COM-B potential specific action or recommendations.

They used a rigorous scoping review approach following JBI and PRISMA‑ScR guidelines, which strengthens transparency and reproducibility. Scoping reviews inherently map existing literature rather than appraise study quality, limiting conclusions about causal relationships. Their search strategy across multiple databases was comprehensive, but excluded non‑English and grey literature which may introduced language or publication bias (appropriately commented on in the discussion). Although the review systematically extracted and coded facilitators and barriers using the COM‑B framework, the included studies were highly heterogeneous in design, populations, and data quality—and none directly measured digital health literacy—meaning the synthesis relies on indirect evidence that may not reflect true DHL constructs.

Specific comments:

Age is a critical factor in digital literacy and relevant to expected types of digital approaches used. While the authors discuss access to devices and internet in general, they do not distinguish between personal versus shared access (common e.g. with parents for younger aged kids), nor do they explore the impact of shared device dynamics on privacy, autonomy, or effective use of digital tools. This would be useful to pull from the studies or highlight if it is a gap.

The most significant finding emerging from this scoping review, in my opinion, is the striking absence of studies that directly assess digital health literacy (DHL). However, despite highlighting this deficit, the manuscript does not offer clear operational recommendations for how DHL should be measured going forward. Providing guidance on feasible, developmentally appropriate assessment strategies or identifying candidate tools would help translate this important observation into actionable direction for researchers and clinicians. In fact, without determining the level of DHL, all of the posed facilitators and barriers appear presumptive rather than evidence-based, since they could not have been associated with a change in level (ether positive or negative without first assessing the level of DHL).

The ’themes’ to emerge are therefor speculative factors that other authors have posed may be important, but have not shown they are. These will ultimately need to be backed by studies with data.

3. Discussion – I find this statement T”his ScR addressed this gap by synthesizing existing evidence to identify the key psychosocial and accessibility factors that influence DHL for the childhood rheumatic disease population.” to be problematic as how does tallying posed facilitators and barriers fill the gap of negligible data on DHL levels to begin with?

The grammar and spelling were generally good. Note inconsistent hyphens - “face-to-face health care” vs. “face to face health care” “non-DH” vs. “non DH”. Some sentences were long and could be broken up for clarity. They perhaps over-use uncommon abbreviations (PRD, ScR, PCC etc.)

Minor points

Abstract: I find this vague or confusing: “Key facilitators of and barriers to DHL include ability to utilize digital health services, health care provider recommendations, and provider level supports.” Can they authors make a more informative statement in the abstract?

Conclusion:

I’m struggling with the authors’ own reported contradiction that DHL was not assessed, yet the conclusion is that because facilitators and barriers were reported, that “DHL was still found to be a critical facilitator or barrier.”

It also makes this concluding statement: “The results of this ScR indicate that the intended positive outcomes of DH interventions may not be actualized if the DHL of children and adolescents with PRD and their caregiver is not accounted for at the early stages of program planning.” sound more like a pre-existing author belief or assumption than an actual finding of the scoping review data.

If in stating “We believe this review provides a foundational understanding for future research and informs the development of targeted interventions.” by identifying the gap in literature, this is acceptable. However, it would be more ‘useful’ to readers if these authors provided guidance on how DHL levels should be assessed in future studies.

I found the ScR methods sound, and the mapping to COM-B as useful exercise/approach. But I’m not sure how the findings can be used other than to promote and guide improved (needed) research in this area.

**********

what does this mean?). If published, this will include your full peer review and any attached files.

**Do you want your identity to be public for this peer review?**  If you choose “no”, your identity will remain anonymous but your review may still be made public.

For information about this choice, including consent withdrawal, please see our Privacy Policy

Reviewer #1: No

Reviewer #2: No

**********

**Figure resubmission:**

**Reproducibility:**

To enhance the reproducibility of your results, we recommend that authors of applicable studies deposit laboratory protocols in protocols.io, where a protocol can be assigned its own identifier (DOI) such that it can be cited independently in the future. Additionally, PLOS ONE offers an option to publish peer-reviewed clinical study protocols. Read more information on sharing protocols at https://plos.org/protocols?utm_medium=editorial-email&utm_source=authorletters&utm_campaign=protocols

# Author comment: Author response to Decision Letter 1

**DOI:** 10.1371/journal.pdig.0001777.r002

5 May 2026

Attachment Submitted filename: PDIG-D-25-00826-Response to Reviewers.docx

# Peer review: Decision Letter 1

**DOI:** 10.1371/journal.pdig.0001777.r003

**Authors:** Mengyu Wang

15 Jul 2026

Response to Reviewers Revised Manuscript with Track Changes Manuscript**Journal Requirements:** **Additional Editor Comments (if provided):** **Reviewers' Comments:**

**Comments to the Author**

Reviewer #1: (No Response)

**********

publication criteria?

Reviewer #1: Yes

**********

3. Has the statistical analysis been performed appropriately and rigorously?

Reviewer #1: Yes

**********

4. Have the authors made all data underlying the findings in their manuscript fully available (please refer to the Data Availability Statement at the start of the manuscript PDF file)?

The PLOS Data policy

Reviewer #1: Yes

**********

5. Is the manuscript presented in an intelligible fashion and written in standard English?

Reviewer #1: Yes

**********

Reviewer #1: Thank you for the opportunity to review the revised manuscript. Overall, I appreciate the authors’ thoughtful engagement with the feedback. The manuscript has been substantially strengthened through the revisions, particularly with respect to methodological transparency, conceptual framing, and discussion of implications. I offer the following comments for consideration to further strengthen the manuscript prior to acceptance.

1. In the Abstract, the authors state that “all the included studies failed to recognize the concept and impact of DHL.” This framing appears somewhat inconsistent with the rationale presented in the Introduction, which acknowledges that studies may address components of DHL without explicitly using the term itself. Given that the review is based on identifying and synthesizing DHL-related concepts from studies that did not directly measure or name DHL, I encourage the authors to consider different language (e.g., “did not explicitly address” or “did not directly examine”) rather than suggesting that studies “failed” to recognize the concept.

2. The Results section of the Abstract identifies psychological capability and physical opportunity as the dominant COM-B domains but then presents key findings without clearly linking them back to these domains. The authors may wish to clarify how factors such as the ability to utilize digital health services, provider recommendations, and provider-level supports map onto the COM-B framework, or alternatively restructure the sentence so these relationships are more apparent. Similarly, the Abstracts conclusion introduces specific themes related to design, functionality, language, provider supports, and DHL education that are not fully reflected in the Results summary. Consider either expanding the Results section to include these themes first.

3. a. The statement that the lack of DHL acknowledgement in the included studies limits the generalizability and clinical applicability of the evidence is an important observation but may benefit from additional nuance. As the authors note elsewhere, there remains no universally accepted or standardized definition of DHL. As such, the absence of explicit DHL terminology may reflect several possibilities, including a lack of conceptual consensus, differences in terminology, or a true gap in awareness of the construct. Distinguishing between these possibilities would strengthen the discussion and provide a more balanced interpretation of the findings.

b. Relatedly, although the search strategy extended back to 1974, all included studies appear to have been published between 2006 and 2025. This is an interesting observation that may warrant brief discussion, as it suggests that concepts related to DHL in pediatric rheumatic disease have emerged relatively recently alongside the increasing integration of digital technologies into healthcare. Highlighting this trend could provide useful context for readers.

4. In their response to reviewers, the authors note that the literature spans diverse global regions, making broad conclusions regarding environmental and sociocultural influences on DHL challenging. However, it may also be worth considering that the evidence base appears to be concentrated within a relatively small number of predominantly high-income countries, including Canada, the United States, the United Kingdom, the Netherlands, and Australia. This concentration may limit understanding of how broader structural determinants, including digital infrastructure, health system organization, funding models, language diversity, and technology access, influence DHL across different settings. Additional reflection on how the geographic distribution of the evidence may affect the transferability and generalizability of the findings would strengthen the manuscript.

5. The manuscript proposes a novel definition of DHL and subsequently uses the review findings to support its effectiveness, as described in the Discussion. While this is an interesting conceptual contribution, the argument currently risks appearing somewhat circular. I encourage the authors to present the proposed definition as a conceptual framework or hypothesis-generating contribution that may inform future research, rather than as a validated definition of DHL.

6. The finding that rural residence emerged as a facilitator of DHL is both unexpected and noteworthy. Given that this finding appears to contrast with broader literature on rural health disparities, I encourage the authors to engage with it in greater depth. Offering potential explanations, such as greater reliance on digital health due to reduced access to in-person services, increased familiarity with virtual care, or selection effects within the included studies, would strengthen the discussion and help identify important avenues for future research and/or the implications section.

Minor Comments

1. Line 121: Consider using “COM-B Model” rather than “COM-B model” to maintain consistency with terminology used elsewhere in the manuscript.

2. In a few places, the manuscript appears to alternate between the terms “metaliterary” and “metaliteracy” when describing DHL. The authors may wish to review these instances and ensure consistent terminology throughout. If “metaliteracy” is intended, this term may be preferable, as it is an established concept within the information literacy and digital literacy literature, whereas “metaliterary” is less commonly used in this context.

3. Lines 415–418: There appears to be a grammatical issue resulting from the merging of two ideas into a single sentence. Consider revising this section to either remove the phrase “are required” or divide the passage into two separate sentences for clarity.

**********

what does this mean?). If published, this will include your full peer review and any attached files.

**Do you want your identity to be public for this peer review?**  If you choose “no”, your identity will remain anonymous but your review may still be made public.

For information about this choice, including consent withdrawal, please see our Privacy Policy

Reviewer #1: No

**********

**Figure resubmission:**

**Reproducibility:**

To enhance the reproducibility of your results, we recommend that authors of applicable studies deposit laboratory protocols in protocols.io, where a protocol can be assigned its own identifier (DOI) such that it can be cited independently in the future. Additionally, PLOS ONE offers an option to publish peer-reviewed clinical study protocols. Read more information on sharing protocols at https://plos.org/protocols?utm_medium=editorial-email&utm_source=authorletters&utm_campaign=protocols

# Author comment: Author response to Decision Letter 2

**DOI:** 10.1371/journal.pdig.0001777.r004

11 Aug 2026

Attachment Submitted filename: PDIG-D-25-00826-Response to Reviewers Aug 10.docx

# Peer review: Decision Letter 2

**DOI:** 10.1371/journal.pdig.0001777.r005

**Authors:** Mengyu Wang

24 Sep 2026

Identifying Facilitators of and Barriers to Digital Health Literacy in Pediatric Rheumatic Disease: A Scoping Review

PDIG-D-25-00826R2

Dear Mr. Eling,

We are pleased to inform you that your manuscript 'Identifying Facilitators of and Barriers to Digital Health Literacy in Pediatric Rheumatic Disease: A Scoping Review' has been provisionally accepted for publication in PLOS Digital Health.

Before your manuscript can be formally accepted you will need to complete some formatting changes, which you will receive in a follow-up email from a member of our team.

Please note that your manuscript will not be scheduled for publication until you have made the required changes, so a swift response is appreciated.

IMPORTANT: The editorial review process is now complete. PLOS will only permit corrections to spelling, formatting or significant scientific errors from this point onwards. Requests for major changes, or any which affect the scientific understanding of your work, will cause delays to the publication date of your manuscript.

If your institution or institutions have a press office, please notify them about your upcoming paper to help maximize its impact. If they'll be preparing press materials, please inform our press team as soon as possible -- no later than 48 hours after receiving the formal acceptance. Your manuscript will remain under strict press embargo until 2 pm Eastern Time on the date of publication. For more information, please contact digitalhealth@plos.org.

Thank you again for supporting Open Access publishing; we are looking forward to publishing your work in PLOS Digital Health.

Best regards,

Mengyu Wang, Ph.D.

Section Editor

PLOS Digital Health

***********************************************************

**Additional Editor Comments (if provided):**

**Reviewer Comments (if any, and for reference):**

Reviewer's Responses to Questions

**Comments to the Author**

Reviewer #1: All comments have been addressed

**********

publication criteria?

Reviewer #1: Yes

**********

3. Has the statistical analysis been performed appropriately and rigorously?

Reviewer #1: N/A

**********

4. Have the authors made all data underlying the findings in their manuscript fully available (please refer to the Data Availability Statement at the start of the manuscript PDF file)?

The PLOS Data policy

Reviewer #1: Yes

**********

5. Is the manuscript presented in an intelligible fashion and written in standard English?

Reviewer #1: Yes

**********

Reviewer #1: Thank you for the opportunity to review the article. No further comments.

**********

what does this mean?). If published, this will include your full peer review and any attached files.

**Do you want your identity to be public for this peer review?**  If you choose “no”, your identity will remain anonymous but your review may still be made public.

For information about this choice, including consent withdrawal, please see our Privacy Policy

Reviewer #1: No

**********
